# *Solanum venturii*, a suitable model system for virus-induced gene silencing studies in potato reveals St*MKK6* as an important player in plant immunity

**DOI:** 10.1186/s13007-016-0129-3

**Published:** 2016-05-20

**Authors:** David Dobnik, Ana Lazar, Tjaša Stare, Kristina Gruden, Vivianne G. A. A. Vleeshouwers, Jana Žel

**Affiliations:** Department of Biotechnology and Systems Biology, National Institute of Biology, Večna Pot 111, 1000 Ljubljana, Slovenia; Wageningen UR Plant Breeding, Wageningen University and Research Centre, P.O. Box 386, 6700 AJ Wageningen, The Netherlands

**Keywords:** Potato, Virus-induced gene silencing, VIGS, Potato virus Y, PVY, *Solanum venturii*, St*WIPK*, St*MKK6*, TRV

## Abstract

**Background:**

Virus-induced gene silencing (VIGS) is an optimal tool for functional analysis of genes in plants, as the viral vector spreads throughout the plant and causes reduced expression of selected gene over the whole plant. Potato (*Solanum tuberosum*) is one of the most important food crops, therefore studies performing functional analysis of its genes are very important. However, the majority of potato cultivars used in laboratory experimental setups are not well amenable to available VIGS systems, thus other model plants from *Solanaceae* family are used (usually *Nicotiana benthamiana*). Wild potato relatives can be a better choice for potato model, but their potential in this field was yet not fully explored. This manuscript presents the set-up of VIGS, based on *Tobacco rattle virus* (TRV) in wild potato relatives for functional studies in potato–virus interactions.

**Results:**

Five different potato cultivars, usually used in our lab, did not respond to silencing of phytoene desaturase (PDS) gene with TRV-based vector. Thus screening of a large set of wild potato relatives (different *Solanum* species and their clones) for their susceptibility to VIGS was performed by silencing PDS gene. We identified several responsive species and further tested susceptibility of these genotypes to potato virus Y (PVY) strain NTN and N. In some species we observed that the presence of empty TRV vector restricted the movement of PVY. Fluorescently tagged PVY^N^-GFP spread systemically in only five of tested wild potato relatives. Based on the results, *Solanum venturii* (VNT366-2) was selected as the most suitable system for functional analysis of genes involved in potato–PVY interaction. The system was tested by silencing two different plant immune signalling-related kinases, St*WIPK* and St*MKK6*. Silencing of St*MKK6* enabled faster spreading of the virus throughout the plant, while silencing of *WIPK* had no effect on spreading of the virus.

**Conclusions:**

The system employing *S. venturii* (VNT366-2) and PVY^N^-GFP is a suitable method for fast and simple functional analysis of genes involved in potato–PVY interactions. Additionally, a set of identified VIGS responsive species of wild potato relatives could serve as a tool for general studies of potato gene function.

**Electronic supplementary material:**

The online version of this article (doi:10.1186/s13007-016-0129-3) contains supplementary material, which is available to authorized users.

## Background

Cultivated potato (*Solanum tuberosum* L.) is, after rice, maize and wheat, the world’s fourth most important food crop (http://faostat.fao.org/). Its susceptibility to wide range of pathogens, which diminish its yield, could therefore have a great impact in the food production chain. One of the most important potato pathogens is the *potato virus Y* (PVY). The necrotic isolates of PVY are still responsible for huge agronomic and economic losses [1]. The ability of viruses to cause a disease is determined at the level of molecular interactions between the host plant counterparts and virus factors that can lead to compatible (sensitive) or incompatible (resistant) interactions [2]. The differential sensitivity of potato cultivars lies in their different genetic background, where the resistant ones usually possess *Ry* (extreme resistance) or *Ny* (hypersensitive resistance) gene (reviewed in [3]). However, recent transcriptomic studies revealed the complexity of signalling network involved in the defence response of potato against PVY [4–8].

The use of transient transformation would be a welcome additional tool to evaluate the role of individual genes in potato. Stable genetic transformation is a less preferred approach, since it is a lengthy process, taking at least 6 months to produce substantial number of transformed plants that can be used for functional experiments. However, to study the interaction between potato and PVY, the gene expression must be modified throughout the whole plant and not only locally, as the virus also spreads systemically. Virus-induced gene silencing (VIGS) is an optimal tool for this purpose, as the virus vector spreads throughout the plant and causes reduction in activity of selected gene in the whole plant based on post-transcriptional gene silencing (PTGS) [9].

Until now, several VIGS vectors originating from RNA and DNA viruses were developed [10, 11]. The *Tobacco rattle virus* (TRV) vector is the most widely used due to its wide host range and mild symptoms [12]. Some VIGS studies with potato using TRV vector have been already described [13, 14], however they are not being used as routinely in potato as for example in model plant species *Nicotiana benthamiana*.

Tuber-bearing *Solanum* species that belong to section *Petota*, represent a large pool of potato relatives, potentially suitable as model species. Section *Petota* contains mostly species from North and South Americas [15, 16], e.g. *Solanum bulbocastanum*, *Solanum stoloniferum* and also the cultivated potato *Solanum tuberosum*. The resource was already used by the potato breeders to introduce the desired traits into cultivars [17]. As these are the closest relatives to cultivated potato, they would serve as a good model system with best possible data translation to cultivated potato cultivars. Large database was already constructed with information on phylogeny [18] and *Phytophthora infestans* resistance in wild potato relatives [19], but no information exist on resistance or susceptibility of these species to viruses.

In order to evaluate the potential application of TRV-based VIGS for functional analysis of potato genes we first screened cultivars for their susceptibility to TRV-based VIGS. As none of the cultivars responded to VIGS, we decided to test wild potato relatives as the closest possible model system, which could be used for potato–PVY interaction studies. We screened an assortment of wild potato relatives for their susceptibility to TRV-based VIGS and selected the most responsive species for further evaluation of their response to PVY infection. Finally, we were able to select good candidates for studies of gene function in potato–PVY interaction. To show that the selected system is applicable for functional evaluation of a potato genes, we selected a mitogen-activated protein kinase (MAPK) gene St*WIPK* (*A. thaliana* orthologue At*MAPK3*) and a mitogen-activated protein kinase kinase (MKK), gene St*MKK6*. In several studies the members of *WIPK* family were shown to be involved in wound and pathogen responses [20–24], response to oxidative stress [25, 26], drought response and stomata development [27–30]. *WIPK* was shown to be responsive after the infection of *N. benthamiana* with potato virus X (PVX) and PVY [31]. *MKK* genes were also shown to be involved in regulation of cytokinesis [32–35] in abscisic and salicylic acid signalling [36, 37], in salt stress response [36] and also in response to pathogen infection [37–40]. With the developed methodology, we showed that downregulation (silencing) of St*MKK6* promotes the viral spread, whereas silencing of St*WIPK* did not affect the virus.

## Results and discussion

### VIGS on different potato cultivars

TRV-based VIGS is a wide-spread system for gene silencing, therefore our aim was to check its applicability on all the genotypes we use in PVY–potato interaction studies in our lab (Igor, PW363, Santé, Rywal, NahG-Rywal, Désirée, Désirée Glu-III and NahG-Désirée). They are differently sensitive to PVY (Additional file 1) because of their genetic background. NahG plants were genetically modified to impair salicylic acid (SA) accumulation [7, 41] and Glu-III transgenic Désirée harbours β-1,3-glucanase class III gene under control of 35S promoter [42]. No literature data existed on the susceptibility of the selected cultivars to TRV-based VIGS. Experimental plants were agroinfiltrated with a mixture of pTRV1 and pTRV2:CaPDS, constructs for silencing phytoene desaturase (PDS). When silenced the carotenoid biosynthesis is supressed, which makes plants susceptible to photobleaching [43] that can easily be visualized. *N. benthamiana* plants were used as positive control and after 12 days post agroinfiltration, plants have already started to show the photo-bleaching phenotype, which persisted throughout the experiment. To show that the original pTRV2:CaPDS construct originating from pepper, would work also on other plant species, we have analysed the sequence identity of different PDS genes. The observed nucleotide sequence identity of *Capsicum annuum* PDS was higher than 95 % when compared to *S. tuberosum* or *S. nigrum* PDS and 92.7 % when compared to *N. benthamiana* PDS (Additional file 2). As *N. benthamiana* plants served as positive control and were always showing photo-bleached phenotype, we concluded that pTRV2:CaPDS should also work on *Solanum* species.

Both NahG-Rywal and NahG-Désirée plants developed strong disease symptoms (leaves have dried up and fallen off) already at 10 days post agroinfiltration. Most probably the lack of SA allowed high TRV accumulation, which was devastating for the small plants. None of other cultivars showed any signs of photo-bleaching. Cultivar Igor responded by dropping the infiltrated leaves, what often occurs after PVY inoculation in this cultivar [44], potentially indicating the multiplication of TRV. To our knowledge, there are only two publications presenting data on VIGS in potato using TRV, showing only three cultivars of *S. tuberosum* (GT12297-4, Cara and Pentland Ivory) as susceptible [13, 14]. Noteworthy, Brigneti et al. had applied VIGS to potato plants grown from seeds [13], a system not used in many of the labs, as the propagation of potatoes in tissue culture is required for retaining the desired genotype.

### VIGS and wild potato relatives

**Fig. 1:**
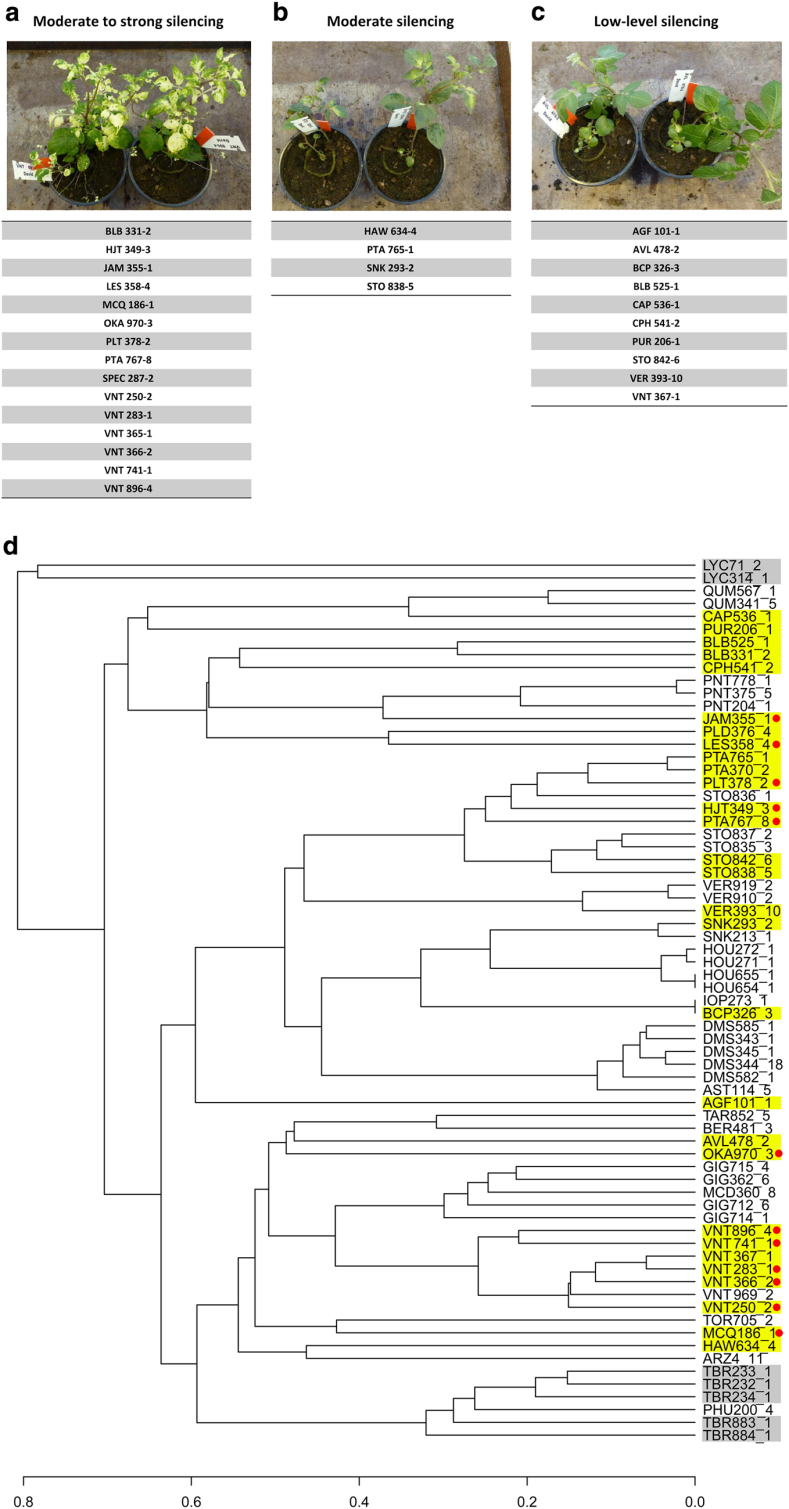
Efficiency of TRV-based VIGS in different wild relatives of potato. The clones that responded to VIGS with TRV (silencing of PDS), were classified into three groups: **a** moderate to strong silencing, **b** moderate silencing and **c** low-level silencing. Photograph at the *top of the panels* **a**–**c** shows silencing phenotype representative for each group. Responsive clones are listed in each corresponding panel. Phylogenetic tree of 62 clones (**d**) that were available in SolRgene database was constructed with interactive online tool using UPGMA method. All VIGS responsive clones are highlighted in *yellow*. The clones from the group of moderate to strong silencing are labelled with a *red dot*. *Solanum lycopersicum* (LYC) and *Solanum tuberosum* (TBR) clones (highlighted in *grey*) were added to the phylogenetic tree to illustrate the relations of wild potato relatives to tomato and cultivated potato. AGF, *Solanum agrimonifolium*; AVL, *Solanum* *avilesii*; BCP, *Solanum brachycarpum*; BLB, *Solanum* *bulbocastanum*; CAP, *Solanum* *capsicibaccatum*; CPH, *Solanum* *cardiophyllum*; HAW, *Solanum* *hawkesianum*; HJT, *Solanum* *hjertingii*; JAM, *Solanum* *jamesii*; LES, *Solanum lesteri*; MCQ, *Solanum* *mochiquense*; OKA, *Solanum* *okadae*; PLT, *Solanum* *polytrichon*; PTA, *Solanum* *papita*; PUR, *Solanum* *piurana*; SNK, *Solanum* *schenckii*; SPEC, *Solanum* *species*; STO, *Solanum* *stoloniferum*; VER, *Solanum verrucosum*; VNT, *Solanum* *venturii*

Brigneti et al. showed that two wild potato relatives, *S. bulbocastanum* and *S. okade*, were susceptible to VIGS [13]. As *S. tuberosum* cultivars tested in our experiments were not responsive to TRV-based VIGS, we have therefore decided to find a set of VIGS susceptible wild potato relatives that could serve as model plants for studies of potato genes. We have tested 73 different clones representing 34 different species of wild potato relatives (Additional file 3), from which all were already tested for resistance/susceptibility to *P. infestans* [19]. Decision to include several clones of the same species was based on observed different resistance level against *P. infestans*. An online phylogenetic analysis to illustrate the relation between *S. tuberosum* and wild potato relatives was conducted within SolRgene [19] with clones that are available in database (Fig. 1). All tested plants were agroinfiltrated with a mixture of pTRV1 and pTRV2:CaPDS and photo-bleaching symptoms were scored 21–24 days post agroinfiltration (dpa). The extent of the recorded photo-bleaching phenotype was classified into four groups: (1) moderate to strong silencing, (2) moderate silencing, (3) low-level silencing and (4) no silencing. 15 clones (10 species) have shown moderate to strong silencing, 4 clones (4 species) have shown moderate silencing and 10 clones (10 species) have shown low-level silencing (Fig. 1). 44 clones (18 species) have shown no silencing (Additional file 3). As the results suggest, the pool of wild potato relatives is not amenable to TRV-based VIGS, with more than half of tested clones being nonresponsive. Even with the overall highly responsive species like *Solanum venturii* or *S. stoloniferum*, there were still some non-responding clones indicating that subtle differences in genotype or epigenetic differences can affect the outcome of plant–TRV interaction. Our results also confirm previous data [13], as *S. bulbocastanum* and *S. okade* species were shown to be in the most responsive group, even though we performed our tests on the plants propagated in stem tissue cultures in contrast to the seed grown plants. When integrating this data with the phylogenetic analysis we observed that at least half of the most responsive clones (mostly *S. venturii*) were relatively closely related to *S. tuberosum* (Fig. 1), when referring to distance and position in a phylogenetic tree in comparison to *S. tuberosum and S. lycopersicon*.

### Susceptibility of selected wild potato relatives to PVY^NTN^

**Fig. 2:**
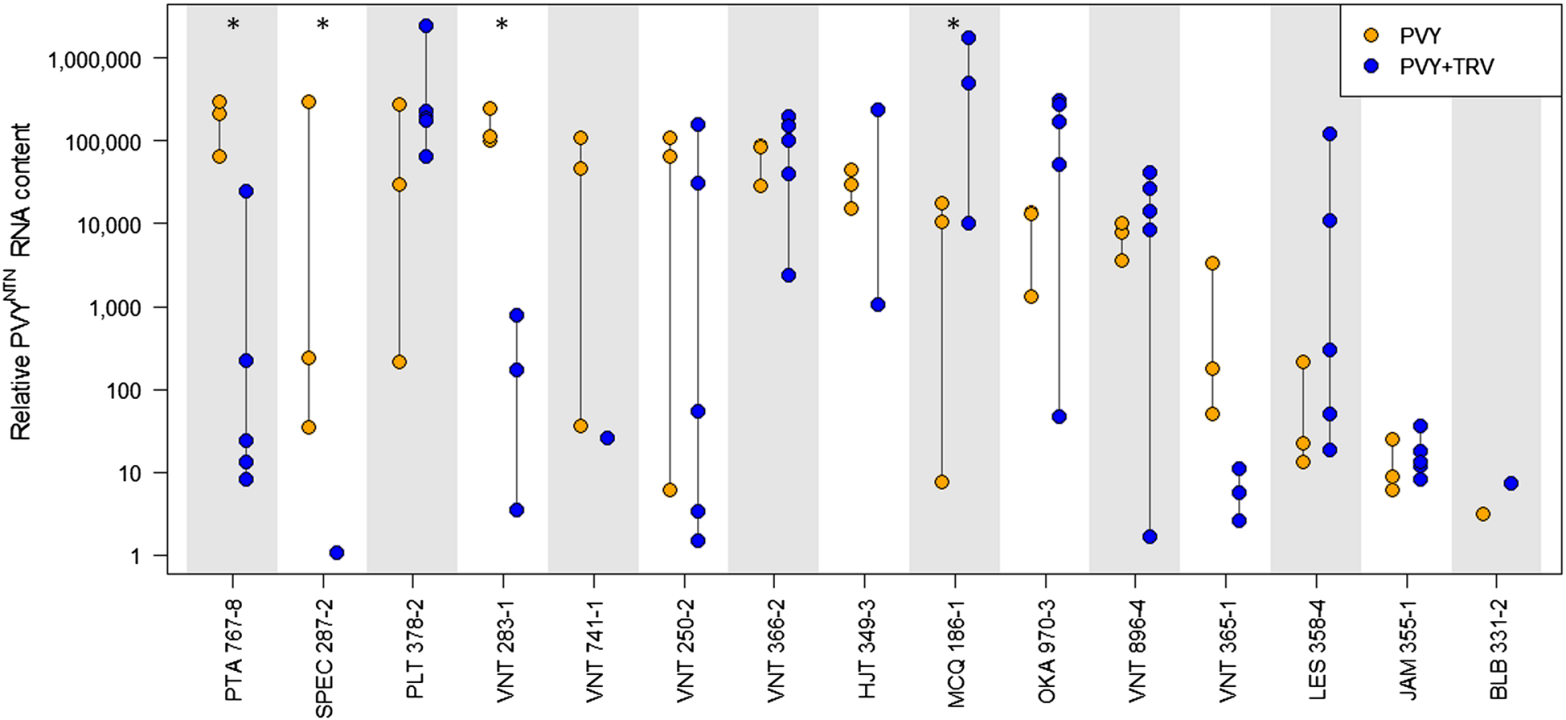
Systemic PVY^NTN^ infection of selected clones. PVY^NTN^ RNA concentration in samples is presented relative to the sample with lowest detected viral amount. Relative PVY^NTN^ RNA content in selected clones 14 dpi after PVY infection (PVY, *orange dots*) and 14 dpi after PVY infection with prior agroinfiltration with empty TRV vector (PVY + TRV, *blue dots*). *Each dot* represents the data from individual plant. Three and five plants were originally infected in PVY and PVY + TRV group, respectively. In the case that the PVY^NTN^ RNA was not detected in some of the samples, the corresponding number of *dots* is lower. *Statistically significant difference in relative PVY^NTN^ RNA content due to agroinfiltration with empty TRV vector (compared between treatments of same genotype). Detailed results of statistical analysis are given in Additional file 5. BLB, *Solanum* *bulbocastanum*; HJT, *Solanum* *hjertingii*; JAM, *Solanum* *jamesii*; LES, *Solanum lesteri*; MCQ, *Solanum* *mochiquense*; OKA, *Solanum* *okadae*; PLT, *Solanum* *polytrichon*; PTA, *Solanum* *papita*; SPEC, *Solanum* *species*; VNT, *Solanum* *venturii*

For the next experiment we selected all 15 clones that were classified as strongly responsive to TRV-based VIGS (Fig. 1a). In order to use the wild potato relatives in functional analysis of genes involved in potato–PVY interaction, their response to PVY must be known. Therefore, the selected clones were inoculated with highly aggressive PVY strain NTN (PVY^NTN^) and we followed its spreading to upper non-inoculated leaves. Samples were collected 14 days post inoculation (dpi), since this is the time when PVY is expected to be present throughout the plant in the case of tolerant interaction [4]. The relative viral RNA content measured in the samples is presented in Fig. 2. All of the tested clones have shown the tolerant phenotype as viral RNA was detected in upper, non-inoculated leaves in all of the clones while no symptoms of viral infection were observed (Additional file 4). *S. bulbocastanum* (BLB 331-2) had the lowest viral RNA content, however we were able to get the data only from 1 out of 3 plants, as the RNA isolation from other two samples was not successful (data not shown). *S jamesii* (JAM 355-1) and *S. lesteri* (LES 358-4) had also relatively low viral content, nevertheless the same low content was observed in individual plants of other clones (Fig. 2) indicating highly variable relative PVY^NTN^ RNA content between plants and genotypes. When observing only the spreading of PVY, the viral content itself is not the true marker for the susceptibility as more susceptible plants don’t necessarily have higher viral content [4]. We showed that there were no significant differences in the amount of viral RNA between clones when infected only with PVY^NTN^ (Additional file 5). Most probably, the variability in the relative amount of PVY^NTN^ RNA is indicating that the time after infection when the virus reaches the upper leaves differs between the individual plants of the same genotype, which was also observed in other experiments studying potato–PVY interaction [4]. These results will be added to the SolRgene database and will complement the information on susceptibility to *P. infestans* in SolRgene database [19].

### The effect of TRV infection on PVY^NTN^ spread in selected wild potato relatives

We showed that PVY^NTN^ spreads in all of the selected clones and in order to use these clones in VIGS studies, we analysed the effect of empty TRV VIGS vector on spreading of PVY^NTN^. As when performing the normal VIGS experiment, we have agroinfiltrated 1 week old plants with empty TRV vector and after 3 additional weeks inoculated the plants with PVY^NTN^. Again, we have tested for presence of PVY^NTN^ RNA in the samples of non-inoculated leaves collected 14 days after PVY^NTN^ inoculation. Although we detected significant differences in PVY^NTN^ content in limited number of cases (Additional file 5), as already discussed above, rather than the relative viral RNA content, the more important indicator of susceptibility to viral infection is the number of plants with detected PVY in upper leaves [42]. In our experiments, prior agroinfiltration with empty TRV vector for VIGS reduced the number of plants successfully infected with PVY^NTN^ (Fig. 2). In three (BLB 331-2, SPEC 287-2 and VNT 741-1) out of seven clones where the reduction in number of infected plants was observed (BLB 331-2, HJT 349-3, MCQ186-1, SPEC 287-2, VNT283-1, VNT 365-1 and VNT 741-1), only one out of five plants was systemically infected.

For functional analysis studies there should be no effect of TRV on the spreading of PVY, therefore we also compared the data of PVY^NTN^ content measured in both experiments (with and without prior TRV agroinfiltration) (Fig. 2, Additional file 5). We showed that the clones suitable for further studies with VIGS, in which the TRV did not affect the spreading of PVY^NTN^, are *S. jamesii* (355-1), *S. lesteri* (358-4), *S. okade* (970-3), *S. polytrichon* (378-2) and *S. venturii* (250-2, 366-2 and 896-4). *S. bulbocastanum*, which was previously already used in TRV-based VIGS experiments for testing resistance genes against *P. infestans* [13], was shown not to be suitable for VIGS in the case of PVY studies, due to the influence of TRV on the course of PVY infection (Fig. 2).

### Susceptibility of selected wild potato relatives to PVY^N^-GFP

**Fig. 3:**
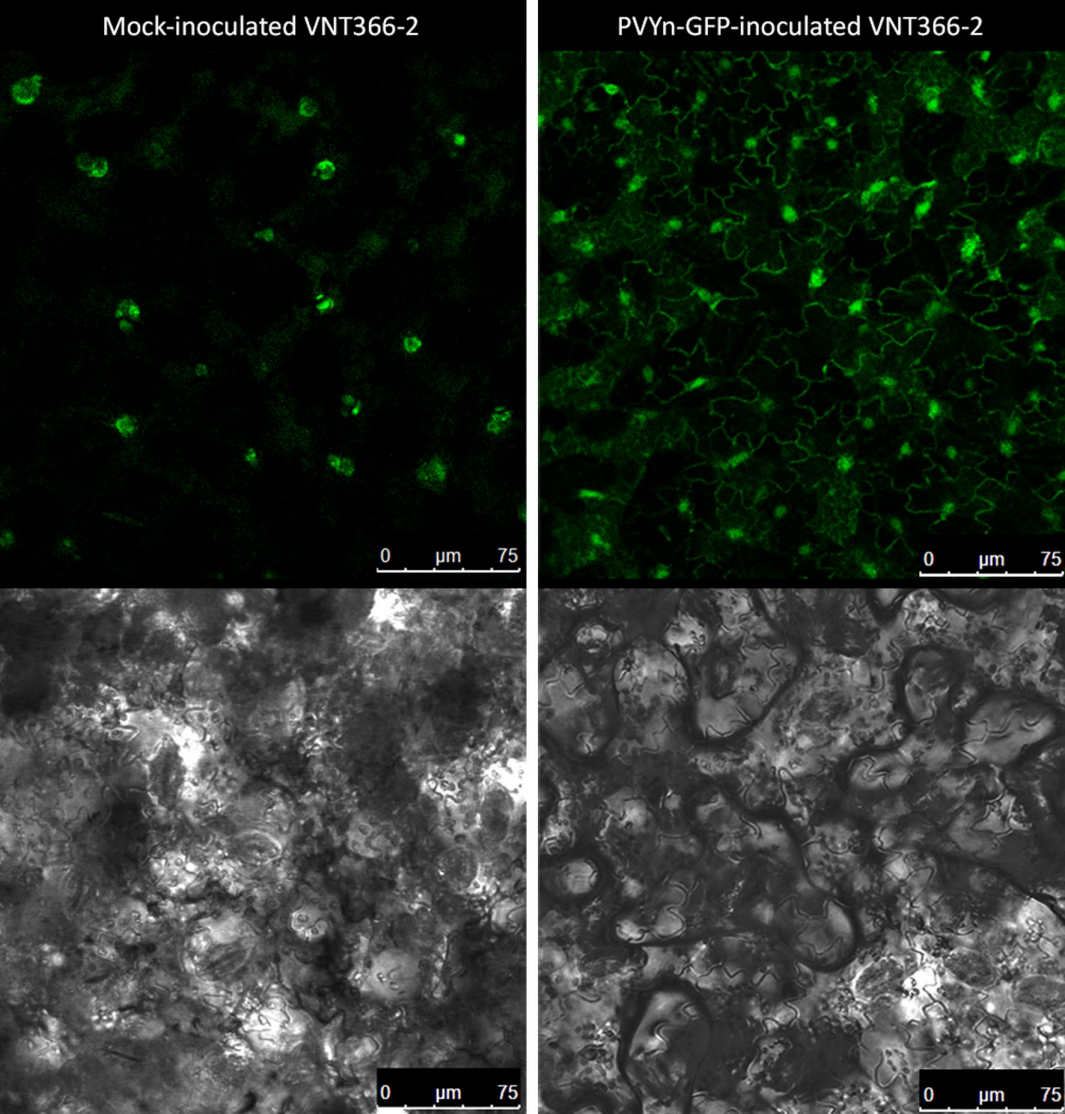
Comparison of green fluorescence signal from mock- and PVY^N^-GFP-inoculated *Solanum venturii* VNT 366-2. Mock inoculated plants can show background green fluorescence signal in a form of clusters (*left side*), whereas the true positive green fluorescence signal for PVY^N^-GFP can be seen as outline of the cells (*right side*), due to the presence of GFP in the cell cytoplasm. *Lower parts* of the figure (*greyscale* images) represent the corresponding transmitted light image of plant leaf section presented in the *top panel* of the figure showing fluorescence signals

Parallel to these studies, an infectious clone of PVY^N^ strain fluorescently labelled with green fluorescent protein (GFP) was developed in our laboratory [45]. Detailed spatio-temporal analyses of viral multiplication are only feasible using fluorescently tagged viruses as then the virus can be monitored in a non-invasive manner using confocal microscopy. In the course of testing the developed infectious clone, also its ability to infect the wild potato relatives was analysed [45]. The green fluorescence, a trace of PVY^N^-GFP multiplication, was detected in a set of TRV-based VIGS responsive wild potato relatives in inoculated leaves (at 14 dpi), indicating the virus was successfully multiplying. On the other hand, GFP fluorescence in non-inoculated leaves was detected at 14 dpi only in five clones (MCQ 186-1, PTA 767-8, VNT 283-1, VNT 365-1 and VNT366-2) [45]. When comparing this report to our results, it is obvious, that the spreading of PVY^N^-GFP is not as efficient as that of PVY^NTN^, since PVY^N^ is less aggressive strain of the PVY [6]. Based on these observations we have performed a more detailed study of PVY^N^-GFP spread in wild potato relatives. When we evaluated the GFP fluorescence in non-inoculated leaves of wild potato relatives at 14 dpi, only VNT 366-2 and VNT283-1 showed the whole area of the non-inoculated leaves covered in green fluorescence and in other three (MCQ 186-1, PTA 767-8 and VNT 365-1) only local patches with green fluorescence were detected. The example of green fluorescence signal in the leaves of VNT 366-2 can be seen in Fig. 3. It is important to mention that some structures exhibiting green fluorescence, most probably due to accumulation of secondary metabolites, can be detected in mock inoculated leaves (Fig. 3), however the green fluorescence pattern can be clearly distinguished from true positive signal (Fig. 3). Finally, VNT 366-2 and VNT 283-1 would be the most suitable candidates for VIGS test subjects, but because in VNT 283-1 the TRV infection interfered with the speed of PVY movement (Fig. 2), we concluded that VNT 366-2 is the most suitable clone for experiments including TRV-based VIGS and PVY^N^-GFP for functional analysis of genes involved in potato–PVY interaction. However, we have also observed that the PVY^N^-GFP was spreading in VNT 366-2 agroinfiltrated with empty TRV with slower rate. The green fluorescence of PVY^N^-GFP in the non-inoculated leaves of an empty vector agroinfiltrated control plants was usually detected at 21 dpi or later (in individual plants green fluorescence was detected at 14 dpi, but never in all biological replicates). Nevertheless, the VIGS system with clone VNT 366-2 is applicable for functional studies of potato genes, even if using milder viral strains.

### Functional confirmation of St*MKK6* role as positive regulator of plant defence against PVY

**Fig. 4:**
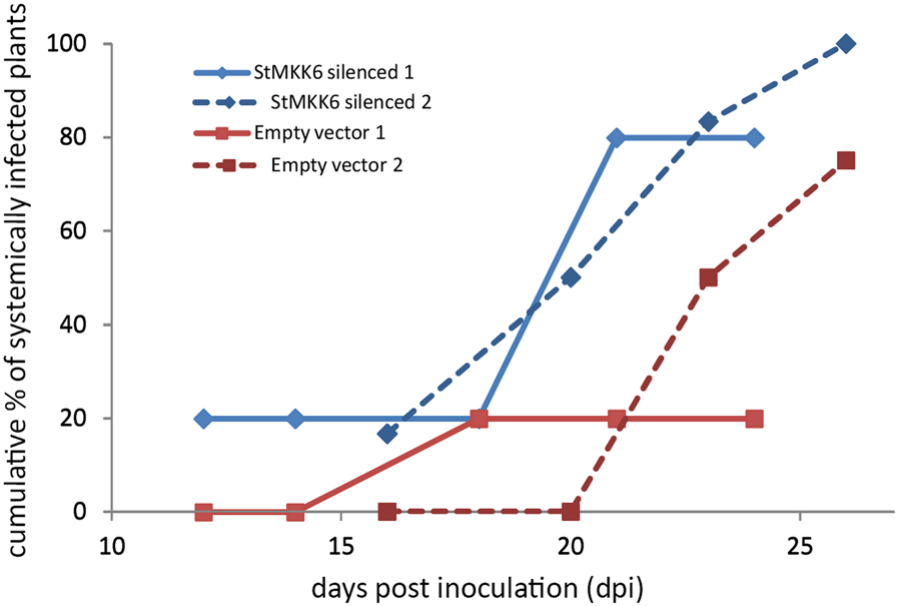
Silencing of St*MKK6* is increasing the speed of viral spread. Cumulative percentage of systemically infected plants after TRV-based VIGS in relation to days post inoculation is shown. The results of two independent time series experiments are presented. In first experiment a total of five biological replicates (plants) was used for each treatment; St*MKK6* silenced (*blue solid line*) and empty TRV vector agroinfiltrated (*red solid line*). In second experiment a total of six biological replicates were used for St*MKK6* silenced group (*blue dashed line*) and four biological replicates for empty TRV vector agroinfiltrated plants (*red dashed line*). At each time point only two or three replicates were analysed

To show the suitability of the selected wild potato relative (clone VNT366-2) for the functional analysis of genes involved in potato–PVY interaction, two different genes from the immune signalling cascade, kinases St*WIPK* and St*MKK6*, were analysed with this system. MAPKs are crucial in plant immune response, therefore disrupting MAPK signalling, by silencing its components, could lead to disturbed plant response to infection with PVY. Both selected genes have been previously reported as important components of plant response to viral infection [31, 37]. We have studied the effect of St*WIPK* and St*MKK6* silencing through observing the GFP fluorescence in the upper, non-inoculated leaves, as a marker of viral spread. Silencing of St*WIPK* had no significant effect on the viral spread in any of the observed time points. The spread of PVY^N^-GFP was also observed in the silenced plants and was comparable to the non-silenced ones (Table 1). There are however several reports on *WIPK* as a positive regulator of immune response [20, 22, 24, 31, 38, 46–49], moreover, in tobacco, silencing of Nt*WIPK* led to reduced resistance to TMV [38, 50]. On the other hand, silencing of St*MKK6* showed that the kinase has important role in viral spread. Emergence of PVY^N^-GFP multiplication marker in non-inoculated leaves of silenced plants preceded the emergence of PVY^N^-GFP in empty vector-treated control plants. Additionally, we detected PVY in *St*MKK6-silenced plants more frequently than in control plants, suggesting that silencing of *St*MKK6 renders plants more susceptible to PVY infection (Fig. 4). These results support our recent findings that St*MKK6* is involved in potato immune response against PVY and acts as positive regulator of potato defence response [37]. It has been shown that *MKK6* gene expression in potato is induced after PVY treatment in a SA-dependant manner [37]. SA is known to influence the expression of defence-related genes [4, 7], especially in response to viral pathogens, through a finely tuned signalling cascades, where many aspects of the regulation still remain undefined [51]. However, based on the results from Arabidopsis, SA signalling regulation with kinases is explained by negative feedback loop, where SA accumulation is negatively regulated by the MAP kinase MPK4 via PAD4 and EDS1 [52, 53]. It was shown that potato St*MKK6* interacts with St*MAPK4* probably through phosphorylation of St*MAPK*4 and additionally that St*MKK6* is involved in HR of potato against PVY a process which is highly dependent on SA [37]. Our results of VIGS, together with our previous findings [37], therefore imply that St*MKK6* is involved in potato immune signalling cascade via interaction with genes involved in SA signalling. Until now, only few reports are available on the topic of *MKK6* silencing. It was shown that silencing of its orthologue has an effect on plant immune response by attenuating the resistance against TMV in *N. tabacum*, most probably through reduced activation of different transcription factors [39]. Moreover, silencing of the orthologue Nb*MEK1* prevented the hypersensitive response (HR) that is triggered via NTF6 cascade [54]. The results of silenced St*MKK6* reported here offer an additional functional confirmation of St*MKK6* as a positive regulator of plant defence against PVY and present additional piece of evidence for the above mentioned connection of SA and St*MKK6*.

**Table 1:** Effect of St*WIPK* silencing on spreading of PVY^N^-GFP to upper non-inoculated leaves

dpi	Systemically infected plants (%)		
	*WIPK* silenced	Empty vector	Positive control
14	0	14.3	33.3
18	0	14.3	nd
22	85.7	85.7	100

dpi, days post inoculation with PVY^N^-GFP; *WIPK* silenced, six plants were agroinfiltrated with TRV construct for silencing *WIPK* gene and infected with PVY^N^-GFP; Empty vector, six plants were agroinfiltrated with empty TRV construct and infected with PVY^N^-GFP; Positive control, three plants were infected with PVY^N^-GFP at the same time as *WIPK* silenced and empty vector plants; nd, not determined at this time point

## Conclusions

We established a fast screening system for evaluation of potato gene function, in particular in response to infection with PVY. As the cultivable potato is not suitable for TRV-based VIGS, we have performed an evaluation of suitability of wild potato relatives for functional analysis of potato genes involved in potato–virus interaction using TRV-based VIGS on a wide set of different species. The most responsive species were further tested for susceptibility to two different PVY strains in order to select the best model species. All tested wild potato relatives were shown to be tolerant to PVY, suggesting they do not possess any resistance genes against this virus. As presented in the manuscript, the system employing *S. venturii* (VNT366-2) and PVY^N^-GFP is the best choice for fast functional analysis of genes. Using the developed system, we have shown that St*MKK6* has a role of positive regulator in potato defence against PVY. Additionally, a set of identified TRV-based VIGS responsive species could also serve as a general tool for functional analyses.

## Methods

### Plant materials and growth conditions

Set of wild potato relatives (wild *Solanum* species, Additional file 3) used in the studies is part of collection of WUR Plant Breeding, Wageningen University and Research Centre, The Netherlands. Different *Solanum tuberosum* cultivars (Additional file 1) are part of the collection of National Institute of Biology, Slovenia. *Nicotiana benthamiana* was used in VIGS experiments as control of silencing. *N. benthamiana* plants were grown from seeds and kept in a growth chamber under controlled conditions (16 h light/8 h dark cycle at 22/20 °C respectively). In vitro potato plantlets of different *Solanum* species were propagated in sterile culture boxes containing MS medium supplemented with 3 % sucrose and 0.8 % agar and grown in a growth chamber under controlled conditions (16 h light/8 h dark cycle at 21/19 °C respectively). Two-week-old plantlets were transplanted into soil and moved to a greenhouse with 22/20 °C day/night temperature regime.

### Plasmid constructs and transformation of *Agrobacterium tumefaciens*

The basic set of TRV VIGS vectors used in the studies was previously described [55]. For PDS silencing we used the pTRV2:CaPDS that is based on pYL156 plasmid backbone with inserted 371-bp fragment (610–980 bp) of PDS gene from *Capsicum annuum* (GenBank accession X68058).

The full-length sequence of St*WIPK* was amplified from *S. tuberosum* cv. Rywal cDNA with the following primers: forward 5-ATGGTTGATGCTAATATGGGT-3 and reverse 5-GCACACAAGCTAGCACGAAC-3. The fragments were inserted into the pJET 1.2 blunt cloning vector (Thermo Scientific) and sequenced (GATC Biotech). For St*WIPK* silencing the 520-bp fragment (187–706 bp) was amplified from pJET plasmid harbouring St*WIPK* gene (GeneBank accession no. KP033231.1) with forward (5′-GAATTCTGAATGAGATGGTTGCAGTT-3′) and reverse (5′-TAAGCTCCATGAAGATGCAA-3′) primer. For St*MKK6* silencing the 453-bp fragment (157–609 bp) was amplified from plasmid harbouring St*MKK6* gene [37] (GeneBank accession no. KF837127.1) with forward (5′-GAATTCTGCCCTCAGAAACTAAGGAG-3′) and reverse (5′-TCCTTTGTGGTTCACTAGCA-3′) primer. In both cases forward primer harboured the EcoRI restriction site. The amplified fragments were inserted into the pJET 1.2 blunt cloning vector (Thermo Scientific) and sequenced (GATC Biotech). pJET plasmids harbouring St*WIPK* or St*MKK6* fragment and empty pTRV2 plasmids were restricted with EcoRI (Gibco) and XbaI (Gibco) restriction enzymes and purified from agarose gel with Wizard SV Gel and PCR Clean-Up System (Promega). Purified fragments were ligated into restricted pTRV2 with T4 DNA ligase (Fermentas). Resulting plasmids pTRV2:WIPK and pTRV2:MKK6 were sequenced (GATC Biotech) and introduced into *Agrobacterium tumefaciens* strain GV3101 by electroporation (Eppendorf Electroporator 2510) following manufacturer’s procedure with voltage set to 2000 V.

### Bioinformatic analysis

Phylogenetic tree of potato and its relatives was prepared with online Interactive phylogeny tool within SolRgene database (http://www.plantbreeding.wur.nl/SolRgenes/Phylogeny/species_select.php). In first step all the species were selected and in the second step the accessions used in our experiments were selected. 11 clones out of 73, which were used in our study for responsiveness to TRV silencing, were not available in the database and are therefore not included in the tree. Additionaly two *S. lycopersicum* and six *S. tuberosum* accessions were added to the phylogenetic tree to illustrate the relations of wild potato relatives to tomato and cultivated potato. A dendrogram [average linkage (UPGMA)] was created.

To check in silico whether the CaPDS construct (part of the sequence with GenBank accession number X68058.1) would have an effect on *Solanum* species, we have aligned different PDS sequences and checked the identity in comparison to CaPDS. The parts of sequences of PDS genes that were used were from *S. tuberosum* (GenBank accession number AY484445.1), *S. nigrum* (GenBank accession number EU434622.1) and *N. benthamiana* (GenBank accession number EU165355.1). Alignment was prepared and analysed with AlignX software (a component of Vector NTI Suite 9.0.0).

### Agroinfiltration for TRV-based VIGS and inoculations with PVY

Plants, after being grown in soil for 7–10 days, were treated by co-infiltration of *Agrobacterium tumefaciens* strain GV3101 carrying pTRV1 and the various pTRV2 recombinants, in a 1:1 ratio as described by Du et al. [56]. pTRV2:CaPDS and empty pTRV2 plasmids were used in initial experiments and later on as controls. For testing VIGS susceptibility of different potato cultivars three plants were tested for each cultivar and for wild potato species two plants were tested for each species.

Five plants of the wild potato relatives were inoculated with buffered suspension of PVY^NTN^ (isolate NIB-NTN, GenBank accession number AJ585342) or PVY^N^-GFP (PVY N605-GFP [45]) infected plant sap 3 weeks after agroinfiltration with empty TRV vector or mock inoculated as described before [8, 45]. For experiments without prior agroinfiltration, three plants of each genotype were inoculated 4 weeks after they were transferred to soil. Samples (upper non-inoculated leaves) for RNA isolation (PVY^NTN^ infected plants) were collected 14 days post inoculation (dpi) and immediately frozen in liquid nitrogen. Samples for confocal microscope observation (PVY^N^-GFP infected plants) were collected 14 dpi and were stored in humid environment (petri dish with humid paper towel) until observed (maximum time from collection to observation was 4 h). For VIGS studies of St*WIPK* six biological replicates were used and four to six biological replicates (details in Additional file 6) for St*MKK6*. Upper non-inoculated leaves were sampled at different time points and observed under confocal microscope. For this purpose, leaves were cut and stored in humid environment until observed as described above.

### Real-time PCR

RNA from the samples was isolated with innuPREP Plant RNA Kit and treated with DNAse (Invitrogen, USA; 0.1 U/Dnase per μg RNA) prior to reverse transcription. 1 μg of RNA was reversely transcribed using the High Capacity cDNA Reverse Transcription Kit (Applied Biosystems, USA).

Samples were analyzed in the set-up for quantitative real-time PCR (qPCR) analysis as previously described [57], using TaqMan chemistry for determining the relative concentration of PVY^NTN^ RNA [58] and cytochrome oxidase (Cox; [59]) as RNA load control. The transcript accumulation was normalized to that of Cox. The relative content of PVY^NTN^ was calculated as follows: Cq value of Cox was subtracted from Cq value for PVY; the resulting value was used as exponential power on value 2; every value was finally divided by the minimum value. The resulting value was transformed with base 10 logarithm. Results were statistically evaluated using Two Way ANOVA in SigmaPlot 13.0 software. Two independent factors were considered in analysis: Genotype (individual species or clones) and Treatment (only PVY infection or PVY infection with prior agroinfiltration with empty TRV vector). The relative PVY RNA content was the dependent variable. For multiple comparison Tukey’s test was selected.

### Confocal microscopy

GFP and background chloroplast fluorescence were visualized with a Leica TCS SP5 laser-scanning microscope mounted on a Leica DMI 6000 CS inverted microscope (Leica Microsystems, Germany) with a HC PL FLUOTAR 10× objective and with Leica TCS LSI macroscope with Plan APO 1× or Plan APO 5× objective (Leica Microsystems, Germany). For excitation, the 488 nm laser line was used. Fluorescence emissions with wavelengths of 505–530 and 590–680 nm were collected simultaneously or sequentially through two channels. Images were processed by using Leica LAS AF Lite software (Leica Microsystems, Germany).

## Additional files

10.1186/s13007-016-0129-3 List of *Solanum tuberosum* cultivars used in this study. All *S. tuberosum* cultivars used in this study are listed together with the description of its susceptibility to PVY or the genetic change for genetically modified lines.
10.1186/s13007-016-0129-3 Sequence alignment of part of PDS gene originating from different species. Through the alignment, the similarity of the part of PDS gene used in VIGS construct to other plant species is presented.
10.1186/s13007-016-0129-3 List of wild potato relatives used in the studies with their response to TRV-based VIGS. All wild potato relatives used in this study are listed together with their abbreviation clone number, country of origin and silencing response.
10.1186/s13007-016-0129-3 Photographs of mock and PVY^NTN^ infected set of wild potato relatives. One mock inoculated (1) and one PVY^NTN^ infected (2) plant at 14 dpi are shown. Letters represent individual species/clones: a) BLB 331-2, b) HJT 349-3, c) JAM 355-1, d) MCQ 186-1, e) OKA 970-3, f) PTA 767-8, g) PLT 378-2, h) SPEC 287-2, i) VNT 250-2, j) VNT 283-1, k) VNT 365-1, l) VNT 366-2, m) VNT 741-1, n) VNT 896-4, o) LES 358-4.
10.1186/s13007-016-0129-3 Report for Two Way ANOVA statistical analysis of relative PVY^NTN^ RNA content using two independent factors. Statistical comparison (Two Way ANOVA) was conducted between groups of plants from individual clones (genotypes) and groups with different treatment (PVY or PVY + TRV). Difference in PVY^NTN^ RNA content is considered statistically significant, if the *P* value is lower than 0.05 (all cases are highlighted in yellow).
10.1186/s13007-016-0129-3 Number of infected plants at each time point in StMKK6 silenced and empty vector treated plants for two different experiments. The table shows the actual number of plants, which were used to calculate cumulative percentage of systemically infected plants after TRV-based VIGS in relation to days post inoculation, as shown in Fig. 4.
