# Supplementary material for: Solanum venturii, a suitable model system for virus-induced gene silencing studies in potato reveals StMKK6 as an important player in plant immunity
Source: Plant Methods. 2016 May 20;12:29. doi: 10.1186/s13007-016-0129-3 (PMC4875682; doi:10.1186/s13007-016-0129-3)
Supplement: Supplementary file 1 — 10.1186/s13007-016-0129-3 List of Solanum tuberosum cultivars used in this study. All S. tuberosum cultivars used in this study are listed together with the description of its susceptibility to PVY or the genetic change for genetically modified lines. [file 13007_2016_129_MOESM1_ESM.pdf]

Additional file 1: List of *Solanum tuberosum* cultivars used in this study

***Solanum tuberosum* cultivar**	**Susceptibility to virus or genetic change**
Igor	Highly susceptible to PVY^{NTN}
PW363	Extremely resistant to PVY^{NTN}
Santé	Extremely resistant to PVY^{NTN}
Rywal	Resistant to PVY^{NTN}
Rywal NahG	Defect in the metabolism of salicylic acid
Désirée	Tolerant to PVY^{NTN}
Désirée Glu-III	Overexpression of β-1,3-glucanase class III
Désirée NahG	Defect in the metabolism of salicylic acid
