# Supplementary material for: Solanum venturii, a suitable model system for virus-induced gene silencing studies in potato reveals StMKK6 as an important player in plant immunity
Source: Plant Methods. 2016 May 20;12:29. doi: 10.1186/s13007-016-0129-3 (PMC4875682; doi:10.1186/s13007-016-0129-3)
Supplement: Supplementary file 2 — 10.1186/s13007-016-0129-3 Sequence alignment of part of PDS gene originating from different species. Through the alignment, the similarity of the part of PDS gene used in VIGS construct to other plant species is presented. [file 13007_2016_129_MOESM2_ESM.pdf]

Additional file 2: Sequence alignment of part of PDS gene originating from different species.

```
                   (1) 1                                                                               83
    CaPDS_construct (1) AATTCAGCCGCTTTGATTTCCCCGAAGCTTTACCTGCTCCTTTAAATGGAATTTTGGCAATCCTAAAGAACAATGAAATGCTT
 N_benthamiana_PDS (1) AGTTCAGCCGCTTTGATTTTCCTGAAGCTCTTCCTGCGCCATTAAATGGAATTTTGGCCATACTAAAGAACAACGAAATGCTT
       S_nigrum_PDS (1) AATTCAGCCGTTTTGATTTCCCCGAAGCTTTACCCGCTCCTTTAAATGGAATTTTGGCCATCCTAAAGAACAATGAAATGCTT
    S_tuberosum_PDS (1) AATTTAGCCGCTTTGATTTCCCCGAAGCTTTACCCGCTCCTTTAAATGGAGTTTTGGCCATCCTAAAGAACAATGAAATGCTT

                  (84) 84                                                                              166
   CaPDS_construct (84) ACATGGCCAGAAAAAGTCAAATTTGCAATTGGACTCTTGCCAGCAATGCTTGGTGGGCAATCTTATGTTGAAGCTCAAGACGG
 N_benthamiana_PDS (84) ACGTGGCCCGAGAAAGTCAAATTTGCTATTGGACTCTTGCCAGCAATGCTTGGAGGGCAATCTTATGTTGAAGCTCAAGACGG
      S_nigrum_PDS (84) ACATGGCCAGAGAAAGTCAAATTTGCAATTGGACTCTTGCCAGCAATGCTTGGAGGGCAATCATATGTTGAAGCTCAAGATGG
   S_tuberosum_PDS (84) ACATGGCCAGAGAAAGTCAAATTTGCAATTGGACTCTTGCCAGCAATGCTTGGAGGGCAATCTTATGTTGAAGCTCAAGACGG

                 (167) 167                                                                             249
  CaPDS_construct (167) GATAAGTGTTAAGGACTGGATGAGAAAACAAGGTGTGCCGGATAGGGTGACGGATGAGGTGTTCATCGCCATGTCAAAGGCAC
N_benthamiana_PDS (167) TTTAAGTGTTAAGGACTGGATGAGAAAGCAAGGTGTGCCTGATAGGGTGACAGATGAGGTGTTCATTGCCATGTCAAAGGCAC
     S_nigrum_PDS (167) GATAAGTGTTAAGGACTGGATGAGAAAGCAAGGTGTGCCGGATAGGGTGACAGATGAGGTGTTCATCGCCATGTCAAAGGCAC
  S_tuberosum_PDS (167) GATAAGTGTTAAGGACTGGATGAGAAAGCAAGGTGTGCCGGATAGGGTGACAGATGAAGTGTTCATCGCCATGTCAAAGGCAC

                 (250) 250                                                                             332
  CaPDS_construct (250) TTAACTTCATAAATCCTGATGAGCTTTCGATGCAGTGCATCTTGATCGCGTTGAACAGATTTCTTCAGGAGAAACATGGTTCA
N_benthamiana_PDS (250) TTAACTTCATAAACCCTGACGAGCTTTCGATGCAGTGCATTTTGATTGCTTTGAACAGATTTCTTCAGGAGAAACATGGTTCA
     S_nigrum_PDS (250) TTAATTTTATAAACCCTGATGAACTTTCAATGCAGTGCATCTTGATCGCATTGAACAGGTTTCTTCAGGAGAAACATGGTTCA
  S_tuberosum_PDS (250) TTAACTTTATAAACCCTGACGAACTGTCAATGCAGTGCATCTTGATCGCATTGAACAGGTTTCTCCAGGAGAAACATGGTTCA

                 (333) 333                                370
  CaPDS_construct (333) AAAATGGCCTTTTTAGATGGTAATCCTCCTGAGAGACT
N_benthamiana_PDS (333) AAAATGGCCTTTTTAGATGGTAACCCTCCTGAGAGACT
     S_nigrum_PDS (333) AAAATGGCCTTTTTAGATGGTAATCCTCCTGAGAGACT
  S_tuberosum_PDS (333) AAAATGGCCTTTTTAGATGGTAATCCTCCTGAGAGACT
```
