# Supplementary material for: Solanum venturii, a suitable model system for virus-induced gene silencing studies in potato reveals StMKK6 as an important player in plant immunity
Source: Plant Methods. 2016 May 20;12:29. doi: 10.1186/s13007-016-0129-3 (PMC4875682; doi:10.1186/s13007-016-0129-3)
Supplement: Supplementary file 3 — 10.1186/s13007-016-0129-3 List of wild potato relatives used in the studies with their response to TRV-based VIGS. All wild potato relatives used in this study are listed together with their abbreviation clone number, country of origin and silencing response. [file 13007_2016_129_MOESM3_ESM.pdf]

Additional file 3: List of wild potato relatives used in the studies with their response to TRV-based VIGS

All wild potato relatives used in this study are listed together with their abbreviation clone number, country of origin and silencing response.

#	Species (*Solanum sp.*)	Abbreviation	CBSG clone	Country of origin	Silencing response
**1**	agrimonifolium	AGF	101-1	Mexico	3
**2**	arnezii	ARZ	4-11	Bolivia	4
**3**	astleyi	AST	114-5	Bolivia	4
**4**	avilesii	AVL	478-2	Bolivia	3
**5**	brachycarpum	BCP	326-3	Mexico	3
**6**	berthaultii	BER	481-3	Bolivia	4
**7**	bulbocastanum	BLB	331-2	Mexico	1
**8**	bulbocastanum	BLB	525-1	Guatemala	3
**9**	capsicibaccatum	CAP	536-1	Bolivia	3
**10**	cardiophyllum	CPH	541-2	Mexico	3
**11**	demissum	DMS	364-1	Peru	4
**12**	demissum	DMS	345-1	Mexico	4
**13**	demissum	DMS	343-1	Mexico	4
**14**	demissum	DMS	344-18	Guatemala	4
**15**	demissum	DMS	585-7	Mexico	4
**16**	demissum	DMS	582-1	Mexico	4
**17**	demissum	DMS	585-1	Mexico	4
**18**	demissum	DMS	299-4	Ecuador	4
**19**	microdontum gigantophyllum	GIG	362-6	Argentina	4
**20**	microdontum gigantophyllum	GIG	712-6	Bolivia	4
**21**	microdontum gigantophyllum	GIG	715-4	Argentina	4
**22**	microdontum gigantophyllum	GIG	714-1	Argentina	4
**23**	hawkesianum	HAW	634-4	Argentina	2
**24**	hjertingii	HJT	349-3	Mexico	1
**25**	hougasii	HOU	654-1	Mexico	4
**26**	hougasii	HOU	655-1	Mexico	4
**27**	hougasii	HOU	272-1	Mexico	4
**28**	hougasii	HOU	271-1	Mexico	4
**29**	iopetalum	IOP	273-1		4
**30**	jamesii	JAM	355-1	USA	1
**31**	lesteri	LES	358-4	Mexico	1
**32**	microdontum	MCD	360-8	Argentina	4
**33**	mochiquense	MCQ	186-1	Peru	1
**34**	okadae	OKA	970-3	Bolivia	1
**35**	phureja	PHU	200-4	Columbia	4
**36**	polyadenium	PLD	376-4	Mexico	4
**37**	polytrichon	PLT	378-2	Mexico	1
**38**	pinnatisectum	PNT	375-5	Mexico	4
**39**	pinnatisectum	PNT	778-1	Mexico	4

**40**	pinnatisectum	PNT	880-3	Mexico	4
**41**	pinnatisectum	PNT	204-1	Mexico	4
**42**	papita	PTA	370-2	Mexico	4
**43**	papita	PTA	767-8	Mexico	1
**44**	papita	PTA	765-1	Mexico	2
**45**	piurana	PUR	206-1	Peru	3
**46**	circaeifolium quimense	QUM	341-5	Bolivia	4
**47**	circaeifolium quimense	QUM	567-1	Bolivia	4
**48**	schenckii	SNK	213-1	Mexico	4
**49**	schenckii	SNK	293-2	Mexico	2
**50**	species	SPEC	891-1	Bolivia	4
**51**	species	SPEC	165-2	Mexico	4
**52**	species	SPEC	210-5	Argentina	4
**53**	species	SPEC	287-2		1
**54**	stoloniferum	STO	842-6	Mexico	3
**55**	stoloniferum	STO	554-2	Peru	4
**56**	stoloniferum	STO	837-2	Mexico	4
**57**	stoloniferum	STO	836-1	Mexico	4
**58**	stoloniferum	STO	838-5	Mexico	2
**59**	stoloniferum	STO	835-3	Mexico	4
**60**	tarijense	TAR	852-5	Bolivia	4
**61**	megistacrolobum toralapanum	TOR	704-5	Bolivia	4
**62**	megistacrolobum toralapanum	TOR	705-2	Bolivia	4
**63**	verrucosum	VER	393-10	Mexico	3
**64**	verrucosum	VER	919-2	Mexico	4
**65**	verrucosum	VER	910-2	Mexico	4
**66**	venturii	VNT	365-1	Argentina	1
**67**	venturii	VNT	367-1	Argentina	3
**68**	venturii	VNT	366-2	Argentina	1
**69**	venturii	VNT	741-1	Argentina	1
**70**	venturii	VNT	250-2	Argentina	1
**71**	venturii	VNT	283-1		1
**72**	venturii	VNT	896-4	Argentina	1
**73**	venturii	VNT	969-2	Argentina	4

Silencing response: 1 - moderate to strong silencing, 2 - moderate silencing, 3 - low-level silencing and 4 - no silencing.
