# Supplementary material for: Solanum venturii, a suitable model system for virus-induced gene silencing studies in potato reveals StMKK6 as an important player in plant immunity
Source: Plant Methods. 2016 May 20;12:29. doi: 10.1186/s13007-016-0129-3 (PMC4875682; doi:10.1186/s13007-016-0129-3)
Supplement: Supplementary file 4 — 10.1186/s13007-016-0129-3 Photographs of mock and PVYNTN infected set of wild potato relatives. One mock inoculated (1) and one PVYNTN infected (2) plant at 14 dpi are shown. Letters represent individual species/clones: a) BLB 331-2, b) HJT 349-3, c) JAM 355-1, d) MCQ 186-1, e) OKA 970-3, f) PTA 767-8, g) PLT 378-2, h) SPEC 287-2, i) VNT 250-2, j) VNT 283-1, k) VNT 365-1, l) VNT 366-2, m) VNT 741-1, n) VNT 896-4, o) LES 358-4. [file 13007_2016_129_MOESM4_ESM.pdf]

Additional file 4: Photographs of mock and PVY^{NTN} infected set of wild potato relatives

One mock inoculated (1) and one PVY^{NTN} infected (2) plant at 14 dpi are shown. Letters represent individual species/clones: a) BLB 331-2, b) HJT 349-3, c) JAM 355-1, d) MCQ 186-1, e) OKA 970-3, f) PTA 767-8, g) PLT 378-2, h) SPEC 287-2, i) VNT 250-2, j) VNT 283-1, k) VNT 365-1, l) VNT 366-2, m) VNT 741-1, n) VNT 896-4, o) LES 358-4.

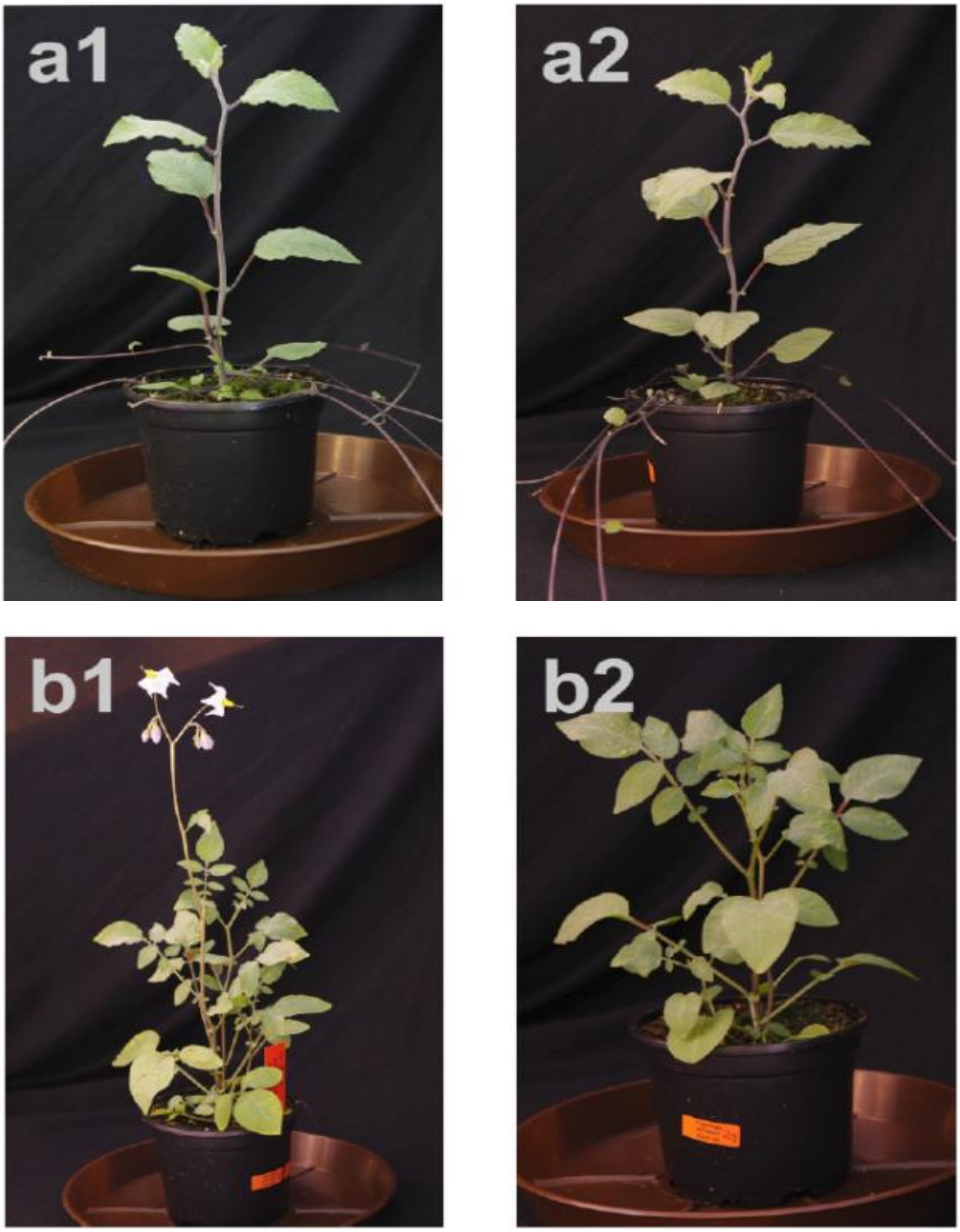

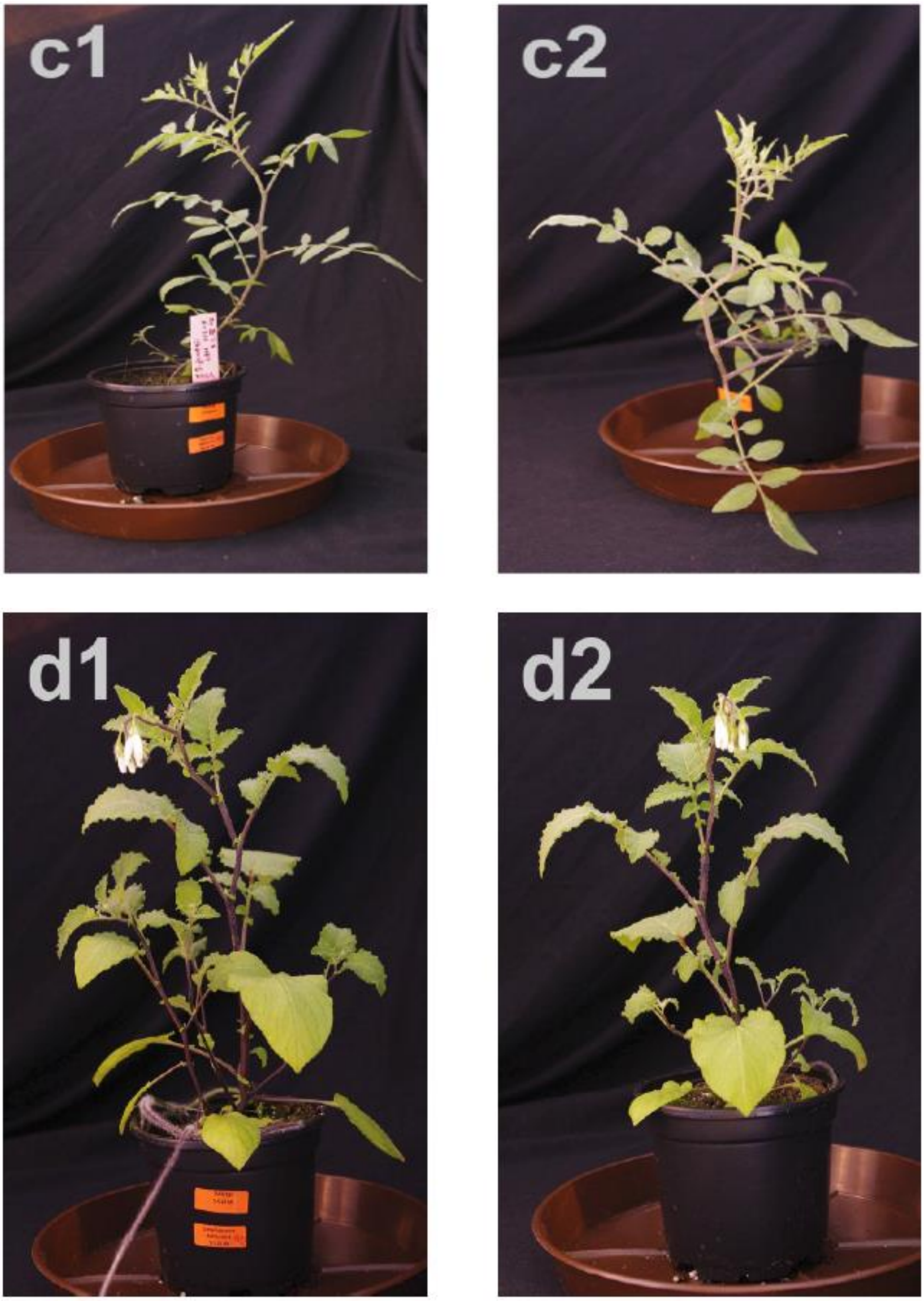
c1
c2
d1
d2

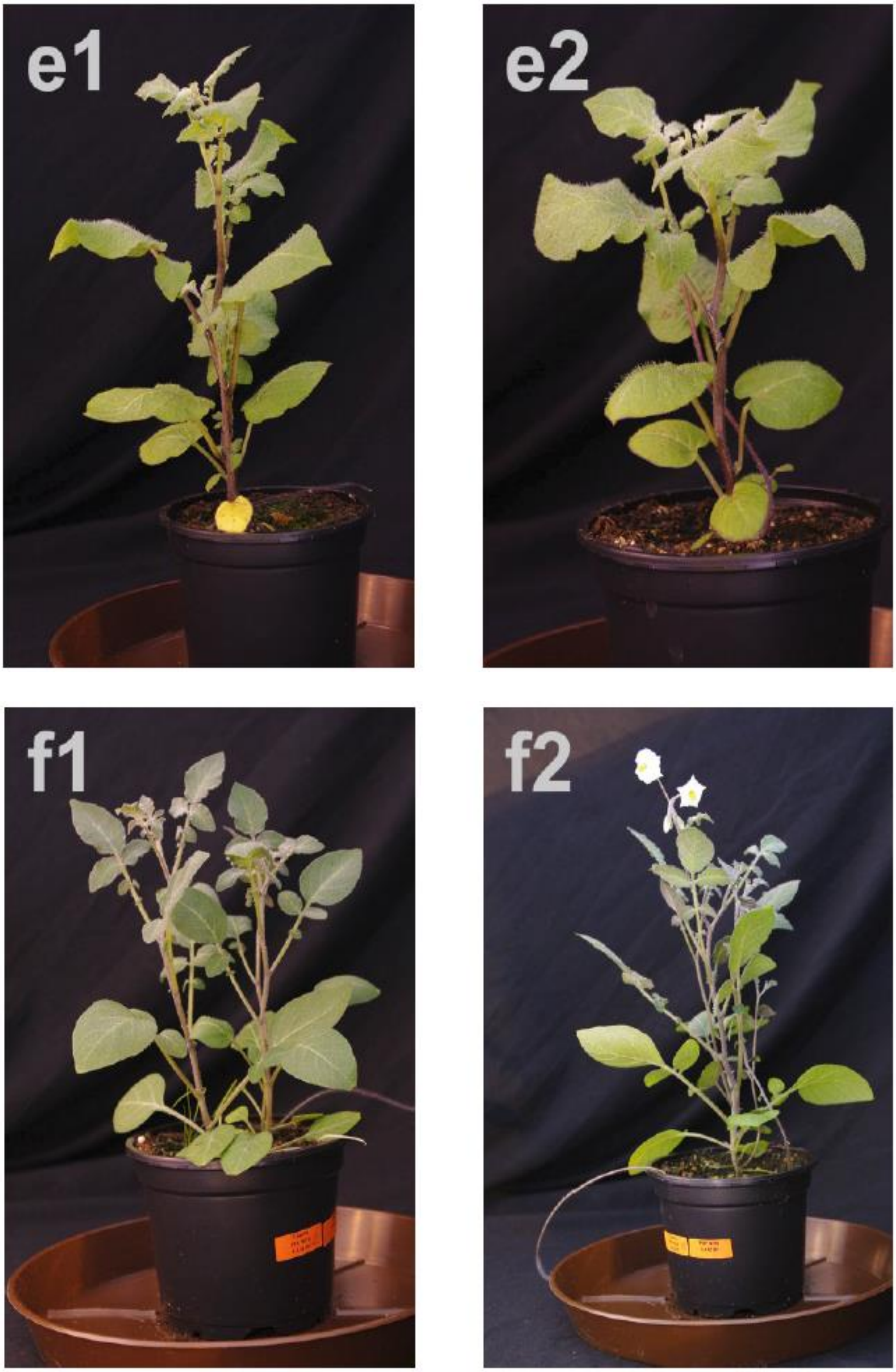
e1
e2
f1
f2

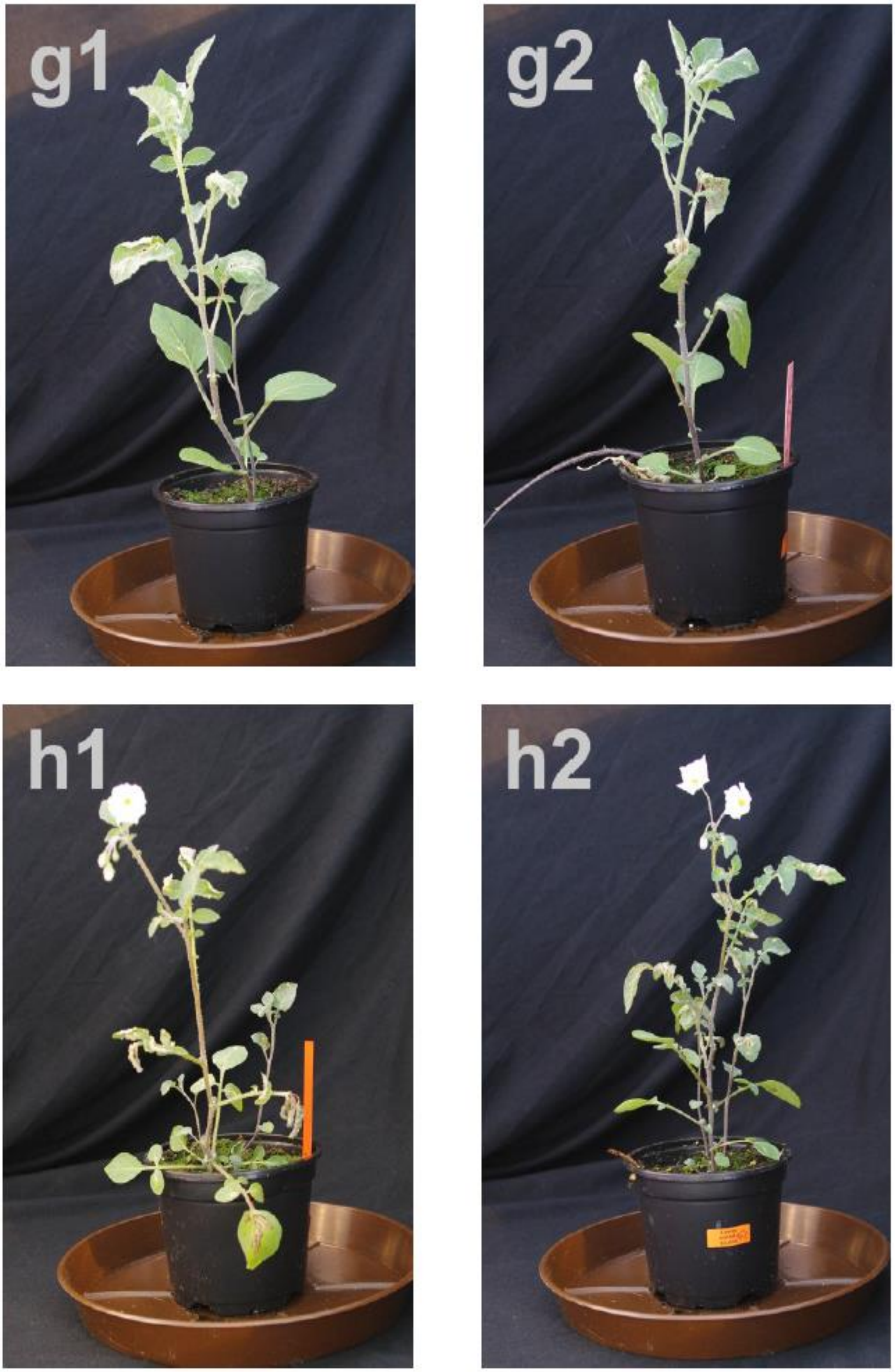
g1
g2
h1
h2

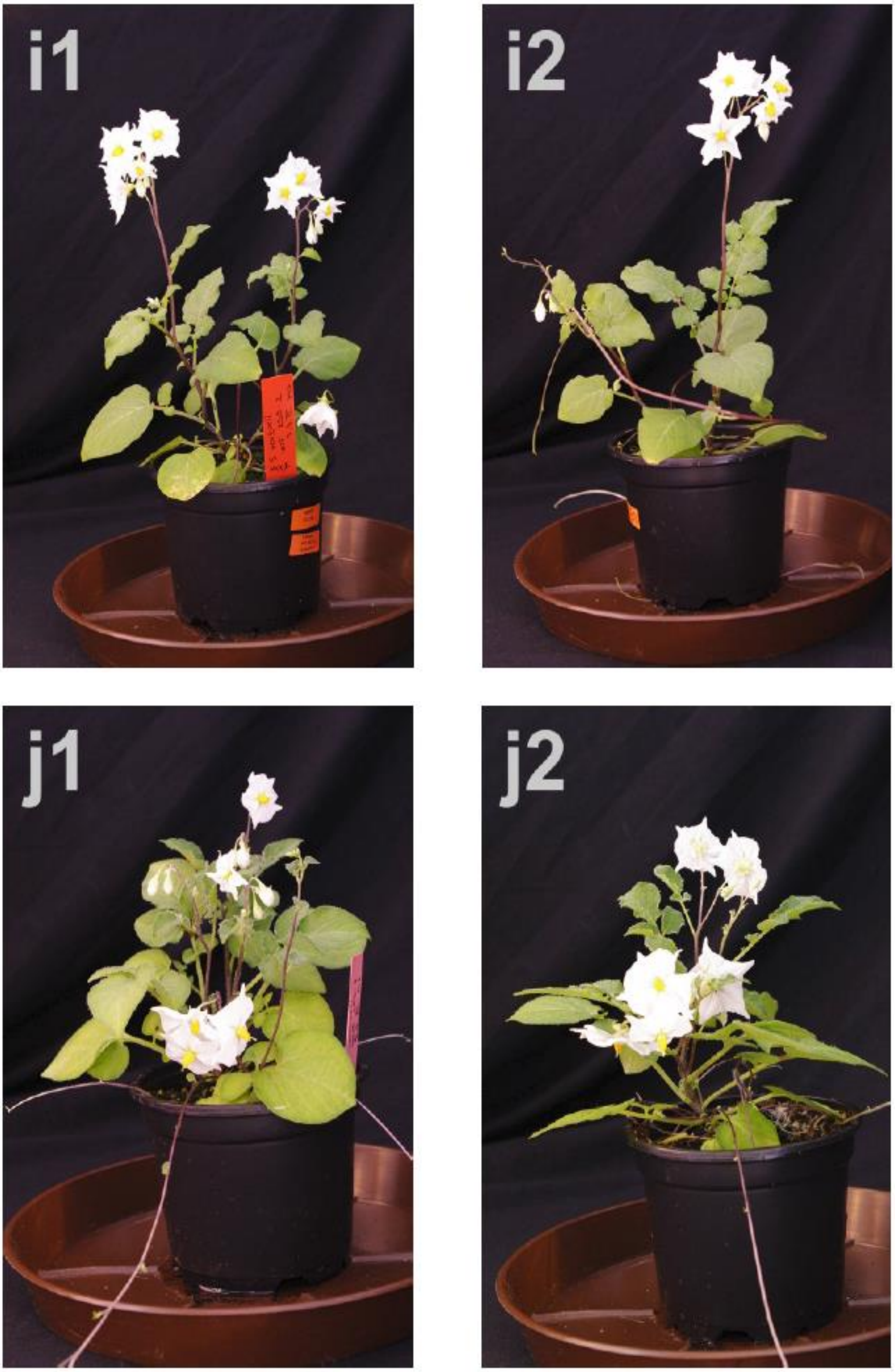
i1
i2
j1
j2

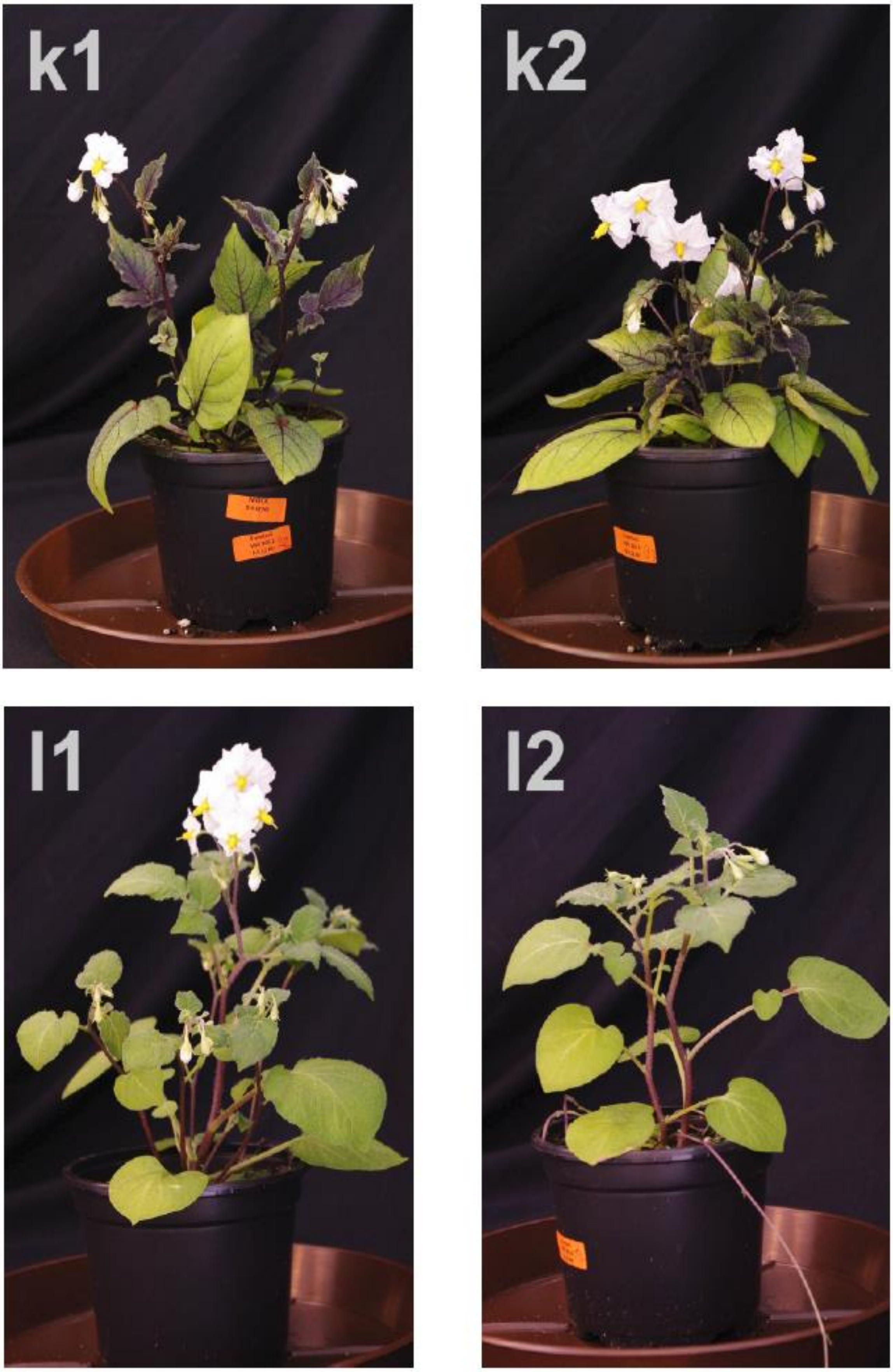
k1
k2
l1
l2

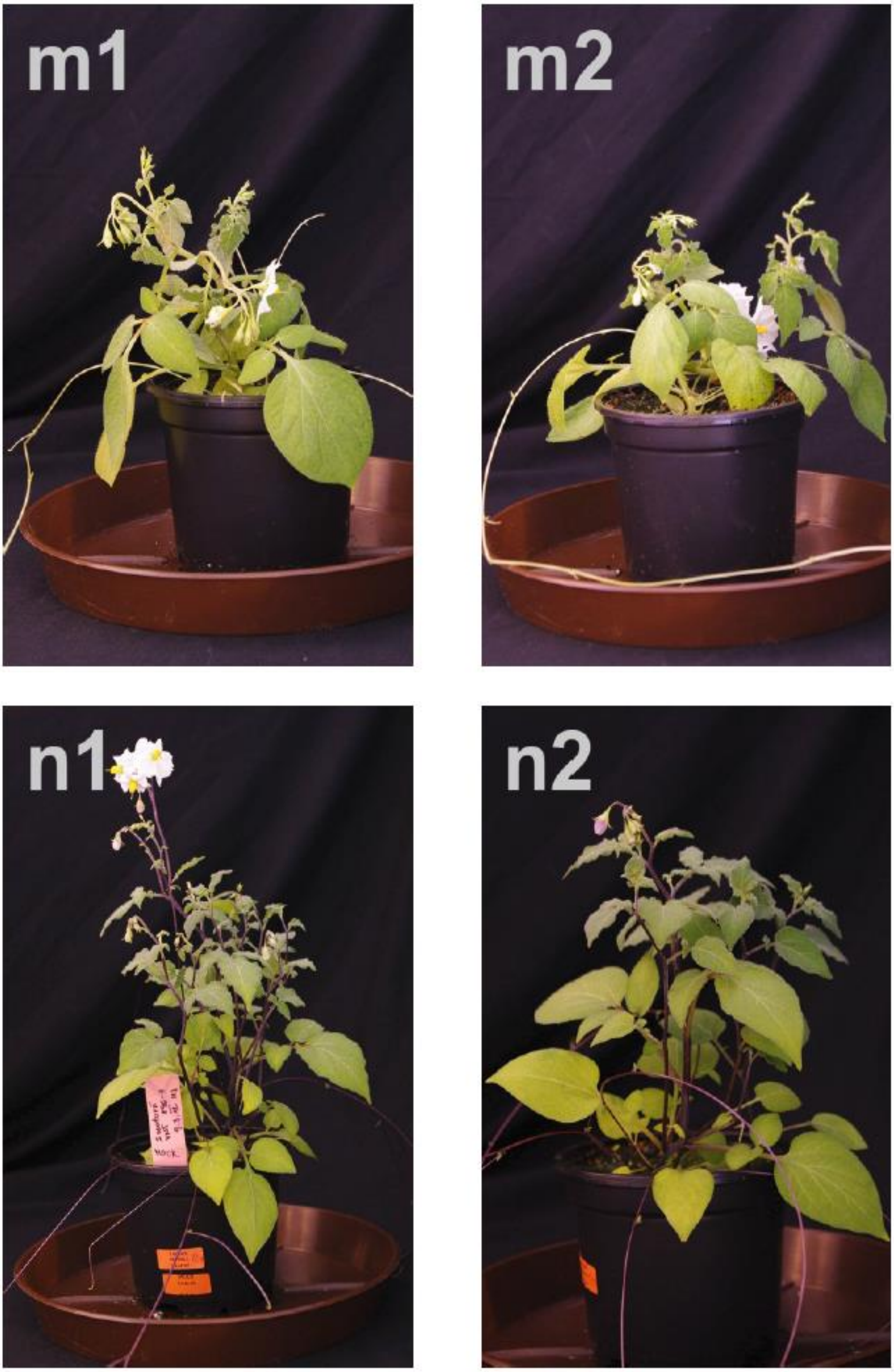
m1
m2
n1
n2

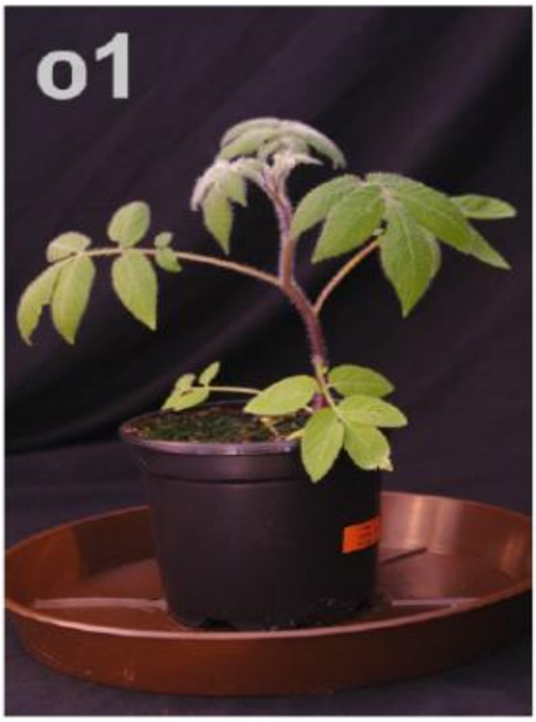
o1

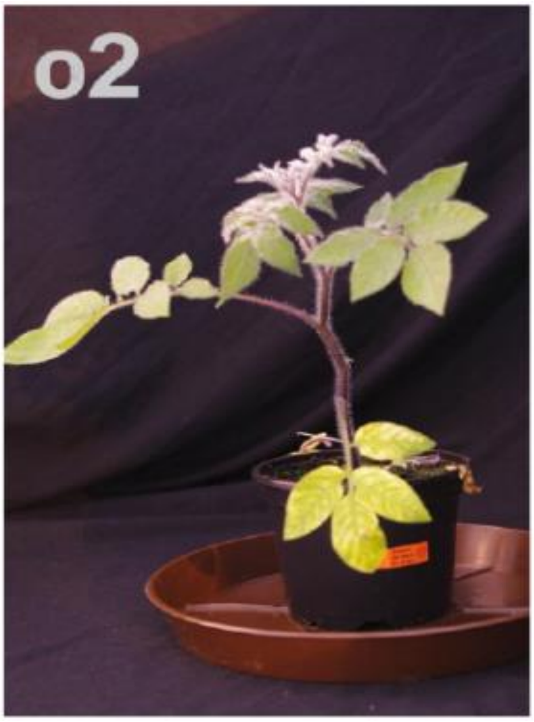
o2
