# Supplementary material for: Solanum venturii, a suitable model system for virus-induced gene silencing studies in potato reveals StMKK6 as an important player in plant immunity
Source: Plant Methods. 2016 May 20;12:29. doi: 10.1186/s13007-016-0129-3 (PMC4875682; doi:10.1186/s13007-016-0129-3)
Supplement: Supplementary file 5 — 10.1186/s13007-016-0129-3 Report for Two Way ANOVA statistical analysis of relative PVYNTN RNA content using two independent factors. Statistical comparison (Two Way ANOVA) was conducted between groups of plants from individual clones (genotypes) and groups with different treatment (PVY or PVY + TRV). Difference in PVYNTN RNA content is considered statistically significant, if the P value is lower than 0.05 (all cases are highlighted in yellow). [file 13007_2016_129_MOESM5_ESM.pdf]

Additional file 5: Report for Two Way ANOVA statistical analysis of relative PVY^{NTN} RNA content using two independent factors.

Two Way Analysis of Variance

General Linear Model

Factor 1: Genotype (different species or clone of wild potato relatives)
Factor 2: Treatment (only PVY infection or PVY infection with prior agroinfiltration with empty TRV vector)
Dependent Variable: logarithmized relative PVY RNA content

Significant differences P<0.05 are highlighted in yellow.

Normality Test (Shapiro-Wilk): Failed (P < 0.050)

Equal Variance Test (Brown-Forsythe): Passed (P = 0.585)

Source of Variation	DF	SS	MS	F	P
Genotype	14	134.047	9.575	5.342	<0.001
Treatment	1	7.309	7.309	4.078	0.047
Genotype x Treatment	14	64.015	4.573	2.551	0.005
Residual	67	120.099	1.793		
Total	96	324.363	3.379		

Main effects cannot be properly interpreted if significant interaction is determined. This is because the size of a factor's effect depends upon the level of the other factor.

The effect of different levels of Genotype depends on what level of Treatment is present. There is a statistically significant interaction between Genotype and Treatment. (P = 0.005)

Power of performed test with alpha = 0.0500: for Genotype : 1.000
Power of performed test with alpha = 0.0500: for Treatment : 0.409
Power of performed test with alpha = 0.0500: for Genotype x Treatment : 0.801

Least square means for Genotype :

Group	Mean	SEM
BLB 331-2	0.677	0.947
HJT 349-3	4.317	0.611
JAM 355-1	1.116	0.489
LES 358-4	2.256	0.489
MCQ 186-1	4.182	0.547
OKA 970-3	4.151	0.489
PLT 378-2	4.753	0.489
PTA 767-8	3.619	0.489
SPEC 287-2	1.577	0.773
VNT 250-2	2.988	0.489
VNT 283-1	3.524	0.547
VNT 365-1	1.612	0.547
VNT 366-2	4.737	0.489
VNT 741-1	2.587	0.773
VNT 896-4	3.645	0.489

Least square means for Treatment :

Group	Mean	SEM
PVY	3.359	0.212
PVY+TRV	2.740	0.220

Least square means for Genotype x Treatment :

Group	Mean	SEM
BLB 331-2 x PVY	0.487	1.339
BLB 331-2 x PVY+TRV	0.867	1.339
HJT 349-3 x PVY	4.436	0.773
HJT 349-3 x PVY+TRV	4.198	0.947
JAM 355-1 x PVY	1.043	0.773
JAM 355-1 x PVY+TRV	1.189	0.599
LES 358-4 x PVY	1.598	0.773
LES 358-4 x PVY+TRV	2.914	0.599
MCQ 186-1 x PVY	3.050	0.773
MCQ 186-1 x PVY+TRV	5.313	0.773
OKA 970-3 x PVY	3.790	0.773
OKA 970-3 x PVY+TRV	4.513	0.599
PLT 378-2 x PVY	4.083	0.773
PLT 378-2 x PVY+TRV	5.423	0.599
PTA 767-8 x PVY	5.208	0.773
PTA 767-8 x PVY+TRV	2.029	0.599
SPEC 287-2 x PVY	3.133	0.773
SPEC 287-2 x PVY+TRV	0.0212	1.339
VNT 250-2 x PVY	3.547	0.773
VNT 250-2 x PVY+TRV	2.428	0.599
VNT 283-1 x PVY	5.156	0.773
VNT 283-1 x PVY+TRV	1.892	0.773
VNT 365-1 x PVY	2.489	0.773

VNT 365-1 x PVY+TRV	0.736	0.773
VNT 366-2 x PVY	4.780	0.773
VNT 366-2 x PVY+TRV	4.694	0.599
VNT 741-1 x PVY	3.756	0.773
VNT 741-1 x PVY+TRV	1.419	1.339
VNT 896-4 x PVY	3.822	0.773
VNT 896-4 x PVY+TRV	3.468	0.599

Comparisons for factor: **Treatment**

**Comparison**	**Diff of Means**	**p**	**q**	**P**	**P<0.050**
PVY vs. PVY+TRV	0.618	2	2.856	0.048	Yes

Comparisons for factor: **Treatment within BLB 331-2**

**Comparison**	**Diff of Means**	**p**	**q**	**P**	**P<0.050**
PVY+TRV vs. PVY	0.380	2	0.284	0.842	No

Comparisons for factor: **Treatment within HJT 349-3**

**Comparison**	**Diff of Means**	**p**	**q**	**P**	**P<0.050**
PVY vs. PVY+TRV	0.238	2	0.275	0.846	No

Comparisons for factor: **Treatment within JAM 355-1**

**Comparison**	**Diff of Means**	**p**	**q**	**P**	**P<0.050**
PVY+TRV vs. PVY	0.145	2	0.210	0.882	No

Comparisons for factor: **Treatment within LES 358-4**

**Comparison**	**Diff of Means**	**p**	**q**	**P**	**P<0.050**
PVY+TRV vs. PVY	1.316	2	1.903	0.183	No

Comparisons for factor: **Treatment within MCQ 186-1**

**Comparison**	**Diff of Means**	**p**	**q**	**P**	**P<0.050**
PVY+TRV vs. PVY	2.263	2	2.928	0.042	Yes

Comparisons for factor: **Treatment within OKA 970-3**

**Comparison**	**Diff of Means**	**p**	**q**	**P**	**P<0.050**
PVY+TRV vs. PVY	0.723	2	1.045	0.463	No

Comparisons for factor: **Treatment within PLT 378-2**

**Comparison**	**Diff of Means**	**p**	**q**	**P**	**P<0.050**
PVY+TRV vs. PVY	1.340	2	1.938	0.175	No

Comparisons for factor: **Treatment within PTA 767-8**

**Comparison**	**Diff of Means**	**p**	**q**	**P**	**P<0.050**
PVY vs. PVY+TRV	3.179	2	4.598	0.002	Yes

Comparisons for factor: **Treatment within SPEC 287-2**

**Comparison**	**Diff of Means**	**p**	**q**	**P**	**P<0.050**
PVY vs. PVY+TRV	3.112	2	2.846	0.048	Yes

Comparisons for factor: **Treatment within VNT 250-2**

Comparison	Diff of Means	p	q	P	P<0.050
PVY vs. PVY+TRV	1.119	2	1.618	0.257	No

Comparisons for factor: **Treatment within VNT 283-1**

Comparison	Diff of Means	p	q	P	P<0.050
PVY vs. PVY+TRV	3.265	2	4.223	0.004	Yes

Comparisons for factor: **Treatment within VNT 365-1**

Comparison	Diff of Means	p	q	P	P<0.050
PVY vs. PVY+TRV	1.753	2	2.268	0.114	No

Comparisons for factor: **Treatment within VNT 366-2**

Comparison	Diff of Means	p	q	P	P<0.050
PVY vs. PVY+TRV	0.0861	2	0.125	0.930	No

Comparisons for factor: **Treatment within VNT 741-1**

Comparison	Diff of Means	p	q	P	P<0.050
PVY vs. PVY+TRV	2.337	2	2.137	0.136	No

Comparisons for factor: **Treatment within VNT 896-4**

Comparison	Diff of Means	p	q	P	P<0.050
PVY vs. PVY+TRV	0.354	2	0.512	0.719	No

All Pairwise Multiple Comparison Procedures (Tukey Test):

Comparisons for factor: **Genotype**

**Comparison**	**Diff of Means**	**p**	**q**	**P**	**P<0.050**
PLT 378-2 vs. BLB 331-2	4.076	15	5.410	0.021	Yes
PLT 378-2 vs. JAM 355-1	3.637	15	7.439	<0.001	Yes
PLT 378-2 vs. SPEC 287-2	3.176	15	4.910	0.057	No
PLT 378-2 vs. VNT 365-1	3.140	15	6.056	0.005	Do Not Test
PLT 378-2 vs. LES 358-4	2.496	15	5.106	0.039	Do Not Test
PLT 378-2 vs. VNT 741-1	2.166	15	3.348	0.546	Do Not Test
PLT 378-2 vs. VNT 250-2	1.765	15	3.610	0.419	Do Not Test
PLT 378-2 vs. VNT 283-1	1.229	15	2.370	0.933	Do Not Test
PLT 378-2 vs. PTA 767-8	1.134	15	2.320	0.943	Do Not Test
PLT 378-2 vs. VNT 896-4	1.107	15	2.265	0.952	Do Not Test
PLT 378-2 vs. OKA 970-3	0.601	15	1.230	1.000	Do Not Test
PLT 378-2 vs. MCQ 186-1	0.571	15	1.101	1.000	Do Not Test
PLT 378-2 vs. HJT 349-3	0.436	15	0.788	1.000	Do Not Test
PLT 378-2 vs. VNT 366-2	0.0157	15	0.0321	1.000	Do Not Test
VNT 366-2 vs. BLB 331-2	4.060	15	5.389	0.022	Yes
VNT 366-2 vs. JAM 355-1	3.621	15	7.407	<0.001	Yes
VNT 366-2 vs. SPEC 287-2	3.160	15	4.886	0.060	Do Not Test
VNT 366-2 vs. VNT 365-1	3.125	15	6.026	0.005	Do Not Test
VNT 366-2 vs. LES 358-4	2.481	15	5.074	0.042	Do Not Test
VNT 366-2 vs. VNT 741-1	2.150	15	3.324	0.558	Do Not Test
VNT 366-2 vs. VNT 250-2	1.749	15	3.578	0.434	Do Not Test
VNT 366-2 vs. VNT 283-1	1.213	15	2.340	0.939	Do Not Test
VNT 366-2 vs. PTA 767-8	1.118	15	2.288	0.949	Do Not Test
VNT 366-2 vs. VNT 896-4	1.092	15	2.233	0.957	Do Not Test
VNT 366-2 vs. OKA 970-3	0.586	15	1.198	1.000	Do Not Test
VNT 366-2 vs. MCQ 186-1	0.555	15	1.071	1.000	Do Not Test
VNT 366-2 vs. HJT 349-3	0.420	15	0.759	1.000	Do Not Test
HJT 349-3 vs. BLB 331-2	3.640	15	4.568	0.106	No
HJT 349-3 vs. JAM 355-1	3.201	15	5.784	0.009	Do Not Test
HJT 349-3 vs. SPEC 287-2	2.740	15	3.932	0.283	Do Not Test
HJT 349-3 vs. VNT 365-1	2.704	15	4.665	0.090	Do Not Test
HJT 349-3 vs. LES 358-4	2.060	15	3.723	0.368	Do Not Test
HJT 349-3 vs. VNT 741-1	1.730	15	2.482	0.907	Do Not Test
HJT 349-3 vs. VNT 250-2	1.329	15	2.402	0.926	Do Not Test
HJT 349-3 vs. VNT 283-1	0.793	15	1.368	1.000	Do Not Test
HJT 349-3 vs. PTA 767-8	0.698	15	1.262	1.000	Do Not Test
HJT 349-3 vs. VNT 896-4	0.672	15	1.214	1.000	Do Not Test
HJT 349-3 vs. OKA 970-3	0.166	15	0.299	1.000	Do Not Test
HJT 349-3 vs. MCQ 186-1	0.135	15	0.233	1.000	Do Not Test
MCQ 186-1 vs. BLB 331-2	3.505	15	4.534	0.113	Do Not Test
MCQ 186-1 vs. JAM 355-1	3.066	15	5.912	0.007	Do Not Test
MCQ 186-1 vs. SPEC 287-2	2.605	15	3.891	0.299	Do Not Test
MCQ 186-1 vs. VNT 365-1	2.569	15	4.701	0.084	Do Not Test
MCQ 186-1 vs. LES 358-4	1.925	15	3.713	0.373	Do Not Test
MCQ 186-1 vs. VNT 741-1	1.594	15	2.382	0.931	Do Not Test

MCQ 186-1 vs. VNT 250-2	1.194	15	2.302	0.946	Do Not Test
MCQ 186-1 vs. VNT 283-1	0.658	15	1.204	1.000	Do Not Test
MCQ 186-1 vs. PTA 767-8	0.563	15	1.086	1.000	Do Not Test
MCQ 186-1 vs. VNT 896-4	0.536	15	1.035	1.000	Do Not Test
MCQ 186-1 vs. OKA 970-3	0.0304	15	0.0586	1.000	Do Not Test
OKA 970-3 vs. BLB 331-2	3.474	15	4.612	0.099	Do Not Test
OKA 970-3 vs. JAM 355-1	3.035	15	6.209	0.004	Do Not Test
OKA 970-3 vs. SPEC 287-2	2.574	15	3.980	0.265	Do Not Test
OKA 970-3 vs. VNT 365-1	2.539	15	4.896	0.059	Do Not Test
OKA 970-3 vs. LES 358-4	1.895	15	3.876	0.304	Do Not Test
OKA 970-3 vs. VNT 741-1	1.564	15	2.418	0.923	Do Not Test
OKA 970-3 vs. VNT 250-2	1.163	15	2.380	0.931	Do Not Test
OKA 970-3 vs. VNT 283-1	0.628	15	1.210	1.000	Do Not Test
OKA 970-3 vs. PTA 767-8	0.533	15	1.090	1.000	Do Not Test
OKA 970-3 vs. VNT 896-4	0.506	15	1.035	1.000	Do Not Test
VNT 896-4 vs. BLB 331-2	2.968	15	3.940	0.280	Do Not Test
VNT 896-4 vs. JAM 355-1	2.529	15	5.174	0.034	Do Not Test
VNT 896-4 vs. SPEC 287-2	2.068	15	3.198	0.621	Do Not Test
VNT 896-4 vs. VNT 365-1	2.033	15	3.920	0.287	Do Not Test
VNT 896-4 vs. LES 358-4	1.389	15	2.841	0.786	Do Not Test
VNT 896-4 vs. VNT 741-1	1.058	15	1.636	0.998	Do Not Test
VNT 896-4 vs. VNT 250-2	0.657	15	1.345	1.000	Do Not Test
VNT 896-4 vs. VNT 283-1	0.121	15	0.234	1.000	Do Not Test
VNT 896-4 vs. PTA 767-8	0.0267	15	0.0545	1.000	Do Not Test
PTA 767-8 vs. BLB 331-2	2.942	15	3.905	0.293	Do Not Test
PTA 767-8 vs. JAM 355-1	2.503	15	5.119	0.038	Do Not Test
PTA 767-8 vs. SPEC 287-2	2.042	15	3.157	0.642	Do Not Test
PTA 767-8 vs. VNT 365-1	2.006	15	3.869	0.307	Do Not Test
PTA 767-8 vs. LES 358-4	1.362	15	2.786	0.808	Do Not Test
PTA 767-8 vs. VNT 741-1	1.031	15	1.595	0.998	Do Not Test
PTA 767-8 vs. VNT 250-2	0.631	15	1.290	1.000	Do Not Test
PTA 767-8 vs. VNT 283-1	0.0948	15	0.183	1.000	Do Not Test
VNT 283-1 vs. BLB 331-2	2.847	15	3.683	0.386	Do Not Test
VNT 283-1 vs. JAM 355-1	2.408	15	4.644	0.093	Do Not Test
VNT 283-1 vs. SPEC 287-2	1.947	15	2.908	0.758	Do Not Test
VNT 283-1 vs. VNT 365-1	1.911	15	3.497	0.473	Do Not Test
VNT 283-1 vs. LES 358-4	1.267	15	2.444	0.917	Do Not Test
VNT 283-1 vs. VNT 741-1	0.937	15	1.399	1.000	Do Not Test
VNT 283-1 vs. VNT 250-2	0.536	15	1.034	1.000	Do Not Test
VNT 250-2 vs. BLB 331-2	2.311	15	3.067	0.685	Do Not Test
VNT 250-2 vs. JAM 355-1	1.872	15	3.829	0.323	Do Not Test
VNT 250-2 vs. SPEC 287-2	1.411	15	2.181	0.965	Do Not Test
VNT 250-2 vs. VNT 365-1	1.375	15	2.652	0.857	Do Not Test
VNT 250-2 vs. LES 358-4	0.731	15	1.496	0.999	Do Not Test
VNT 250-2 vs. VNT 741-1	0.401	15	0.619	1.000	Do Not Test
VNT 741-1 vs. BLB 331-2	1.910	15	2.211	0.961	Do Not Test
VNT 741-1 vs. JAM 355-1	1.471	15	2.275	0.951	Do Not Test
VNT 741-1 vs. SPEC 287-2	1.010	15	1.307	1.000	Do Not Test
VNT 741-1 vs. VNT 365-1	0.975	15	1.456	0.999	Do Not Test
VNT 741-1 vs. LES 358-4	0.331	15	0.511	1.000	Do Not Test

LES 358-4 vs. BLB 331-2	1.580	15	2.097	0.975	Do Not Test
LES 358-4 vs. JAM 355-1	1.141	15	2.333	0.940	Do Not Test
LES 358-4 vs. SPEC 287-2	0.679	15	1.051	1.000	Do Not Test
LES 358-4 vs. VNT 365-1	0.644	15	1.242	1.000	Do Not Test
VNT 365-1 vs. BLB 331-2	0.936	15	1.210	1.000	Do Not Test
VNT 365-1 vs. JAM 355-1	0.496	15	0.957	1.000	Do Not Test
VNT 365-1 vs. SPEC 287-2	0.0354	15	0.0529	1.000	Do Not Test
SPEC 287-2 vs. BLB 331-2	0.900	15	1.042	1.000	Do Not Test
SPEC 287-2 vs. JAM 355-1	0.461	15	0.713	1.000	Do Not Test
JAM 355-1 vs. BLB 331-2	0.439	15	0.583	1.000	Do Not Test

Comparisons for factor: **Genotype within PVY**

**Comparison**	**Diff of Means**	**p**	**q**	**P**	**P<0.050**
PTA 767-8 vs. BLB 331-2	4.721	15	4.319	0.161	No
PTA 767-8 vs. JAM 355-1	4.165	15	5.388	0.022	Do Not Test
PTA 767-8 vs. LES 358-4	3.610	15	4.670	0.089	Do Not Test
PTA 767-8 vs. VNT 365-1	2.719	15	3.518	0.463	Do Not Test
PTA 767-8 vs. MCQ 186-1	2.158	15	2.792	0.806	Do Not Test
PTA 767-8 vs. SPEC 287-2	2.075	15	2.685	0.846	Do Not Test
PTA 767-8 vs. VNT 250-2	1.661	15	2.149	0.969	Do Not Test
PTA 767-8 vs. VNT 741-1	1.453	15	1.879	0.990	Do Not Test
PTA 767-8 vs. OKA 970-3	1.418	15	1.835	0.992	Do Not Test
PTA 767-8 vs. VNT 896-4	1.386	15	1.793	0.994	Do Not Test
PTA 767-8 vs. PLT 378-2	1.125	15	1.456	0.999	Do Not Test
PTA 767-8 vs. HJT 349-3	0.772	15	0.999	1.000	Do Not Test
PTA 767-8 vs. VNT 366-2	0.428	15	0.554	1.000	Do Not Test
PTA 767-8 vs. VNT 283-1	0.0520	15	0.0673	1.000	Do Not Test
VNT 283-1 vs. BLB 331-2	4.669	15	4.271	0.173	Do Not Test
VNT 283-1 vs. JAM 355-1	4.113	15	5.321	0.025	Do Not Test
VNT 283-1 vs. LES 358-4	3.558	15	4.602	0.100	Do Not Test
VNT 283-1 vs. VNT 365-1	2.667	15	3.450	0.496	Do Not Test
VNT 283-1 vs. MCQ 186-1	2.106	15	2.725	0.831	Do Not Test
VNT 283-1 vs. SPEC 287-2	2.023	15	2.617	0.868	Do Not Test
VNT 283-1 vs. VNT 250-2	1.609	15	2.081	0.976	Do Not Test
VNT 283-1 vs. VNT 741-1	1.401	15	1.812	0.993	Do Not Test
VNT 283-1 vs. OKA 970-3	1.366	15	1.767	0.995	Do Not Test
VNT 283-1 vs. VNT 896-4	1.334	15	1.726	0.996	Do Not Test
VNT 283-1 vs. PLT 378-2	1.073	15	1.389	1.000	Do Not Test
VNT 283-1 vs. HJT 349-3	0.720	15	0.932	1.000	Do Not Test
VNT 283-1 vs. VNT 366-2	0.376	15	0.486	1.000	Do Not Test
VNT 366-2 vs. BLB 331-2	4.293	15	3.927	0.285	Do Not Test
VNT 366-2 vs. JAM 355-1	3.737	15	4.834	0.066	Do Not Test
VNT 366-2 vs. LES 358-4	3.182	15	4.116	0.219	Do Not Test
VNT 366-2 vs. VNT 365-1	2.291	15	2.964	0.733	Do Not Test
VNT 366-2 vs. MCQ 186-1	1.730	15	2.238	0.957	Do Not Test
VNT 366-2 vs. SPEC 287-2	1.647	15	2.131	0.971	Do Not Test
VNT 366-2 vs. VNT 250-2	1.233	15	1.595	0.998	Do Not Test
VNT 366-2 vs. VNT 741-1	1.025	15	1.326	1.000	Do Not Test
VNT 366-2 vs. OKA 970-3	0.990	15	1.281	1.000	Do Not Test
VNT 366-2 vs. VNT 896-4	0.958	15	1.239	1.000	Do Not Test
VNT 366-2 vs. PLT 378-2	0.697	15	0.902	1.000	Do Not Test
VNT 366-2 vs. HJT 349-3	0.344	15	0.445	1.000	Do Not Test
HJT 349-3 vs. BLB 331-2	3.949	15	3.612	0.418	Do Not Test
HJT 349-3 vs. JAM 355-1	3.393	15	4.389	0.144	Do Not Test
HJT 349-3 vs. LES 358-4	2.837	15	3.671	0.392	Do Not Test
HJT 349-3 vs. VNT 365-1	1.947	15	2.519	0.897	Do Not Test
HJT 349-3 vs. MCQ 186-1	1.386	15	1.793	0.994	Do Not Test
HJT 349-3 vs. SPEC 287-2	1.303	15	1.686	0.997	Do Not Test
HJT 349-3 vs. VNT 250-2	0.889	15	1.150	1.000	Do Not Test
HJT 349-3 vs. VNT 741-1	0.680	15	0.880	1.000	Do Not Test

HJT 349-3 vs. OKA 970-3	0.646	15	0.836	1.000	Do Not Test
HJT 349-3 vs. VNT 896-4	0.614	15	0.794	1.000	Do Not Test
HJT 349-3 vs. PLT 378-2	0.353	15	0.457	1.000	Do Not Test
PLT 378-2 vs. BLB 331-2	3.596	15	3.289	0.576	Do Not Test
PLT 378-2 vs. JAM 355-1	3.039	15	3.932	0.283	Do Not Test
PLT 378-2 vs. LES 358-4	2.484	15	3.214	0.613	Do Not Test
PLT 378-2 vs. VNT 365-1	1.594	15	2.062	0.978	Do Not Test
PLT 378-2 vs. MCQ 186-1	1.033	15	1.336	1.000	Do Not Test
PLT 378-2 vs. SPEC 287-2	0.950	15	1.229	1.000	Do Not Test
PLT 378-2 vs. VNT 250-2	0.536	15	0.693	1.000	Do Not Test
PLT 378-2 vs. VNT 741-1	0.327	15	0.423	1.000	Do Not Test
PLT 378-2 vs. OKA 970-3	0.293	15	0.379	1.000	Do Not Test
PLT 378-2 vs. VNT 896-4	0.261	15	0.337	1.000	Do Not Test
VNT 896-4 vs. BLB 331-2	3.335	15	3.051	0.693	Do Not Test
VNT 896-4 vs. JAM 355-1	2.779	15	3.595	0.427	Do Not Test
VNT 896-4 vs. LES 358-4	2.224	15	2.877	0.771	Do Not Test
VNT 896-4 vs. VNT 365-1	1.333	15	1.725	0.996	Do Not Test
VNT 896-4 vs. MCQ 186-1	0.772	15	0.999	1.000	Do Not Test
VNT 896-4 vs. SPEC 287-2	0.689	15	0.892	1.000	Do Not Test
VNT 896-4 vs. VNT 250-2	0.275	15	0.356	1.000	Do Not Test
VNT 896-4 vs. VNT 741-1	0.0666	15	0.0861	1.000	Do Not Test
VNT 896-4 vs. OKA 970-3	0.0322	15	0.0416	1.000	Do Not Test
OKA 970-3 vs. BLB 331-2	3.303	15	3.021	0.706	Do Not Test
OKA 970-3 vs. JAM 355-1	2.747	15	3.553	0.446	Do Not Test
OKA 970-3 vs. LES 358-4	2.191	15	2.835	0.788	Do Not Test
OKA 970-3 vs. VNT 365-1	1.301	15	1.683	0.997	Do Not Test
OKA 970-3 vs. MCQ 186-1	0.740	15	0.957	1.000	Do Not Test
OKA 970-3 vs. SPEC 287-2	0.657	15	0.850	1.000	Do Not Test
OKA 970-3 vs. VNT 250-2	0.243	15	0.314	1.000	Do Not Test
OKA 970-3 vs. VNT 741-1	0.0344	15	0.0445	1.000	Do Not Test
VNT 741-1 vs. BLB 331-2	3.268	15	2.990	0.721	Do Not Test
VNT 741-1 vs. JAM 355-1	2.712	15	3.509	0.467	Do Not Test
VNT 741-1 vs. LES 358-4	2.157	15	2.790	0.806	Do Not Test
VNT 741-1 vs. VNT 365-1	1.267	15	1.639	0.998	Do Not Test
VNT 741-1 vs. MCQ 186-1	0.706	15	0.913	1.000	Do Not Test
VNT 741-1 vs. SPEC 287-2	0.623	15	0.805	1.000	Do Not Test
VNT 741-1 vs. VNT 250-2	0.208	15	0.270	1.000	Do Not Test
VNT 250-2 vs. BLB 331-2	3.060	15	2.799	0.803	Do Not Test
VNT 250-2 vs. JAM 355-1	2.504	15	3.239	0.601	Do Not Test
VNT 250-2 vs. LES 358-4	1.949	15	2.521	0.897	Do Not Test
VNT 250-2 vs. VNT 365-1	1.058	15	1.369	1.000	Do Not Test
VNT 250-2 vs. MCQ 186-1	0.497	15	0.643	1.000	Do Not Test
VNT 250-2 vs. SPEC 287-2	0.414	15	0.536	1.000	Do Not Test
SPEC 287-2 vs. BLB 331-2	2.646	15	2.420	0.922	Do Not Test
SPEC 287-2 vs. JAM 355-1	2.090	15	2.703	0.839	Do Not Test
SPEC 287-2 vs. LES 358-4	1.534	15	1.985	0.984	Do Not Test
SPEC 287-2 vs. VNT 365-1	0.644	15	0.833	1.000	Do Not Test
SPEC 287-2 vs. MCQ 186-1	0.0829	15	0.107	1.000	Do Not Test
MCQ 186-1 vs. BLB 331-2	2.563	15	2.345	0.938	Do Not Test
MCQ 186-1 vs. JAM 355-1	2.007	15	2.596	0.875	Do Not Test

MCQ 186-1 vs. LES 358-4	1.451	15	1.878	0.991	Do Not Test
MCQ 186-1 vs. VNT 365-1	0.561	15	0.726	1.000	Do Not Test
VNT 365-1 vs. BLB 331-2	2.002	15	1.831	0.993	Do Not Test
VNT 365-1 vs. JAM 355-1	1.446	15	1.870	0.991	Do Not Test
VNT 365-1 vs. LES 358-4	0.890	15	1.152	1.000	Do Not Test
LES 358-4 vs. BLB 331-2	1.111	15	1.017	1.000	Do Not Test
LES 358-4 vs. JAM 355-1	0.555	15	0.718	1.000	Do Not Test
JAM 355-1 vs. BLB 331-2	0.556	15	0.509	1.000	Do Not Test

Comparisons for factor: **Genotype within PVY+TRV**

**Comparison**	**Diff of Means**	**p**	**q**	**P**	**P<0.050**
PLT 378-2 vs. SPEC 287-2	5.402	15	5.209	0.032	Yes
PLT 378-2 vs. VNT 365-1	4.687	15	6.779	<0.001	Yes
PLT 378-2 vs. BLB 331-2	4.556	15	4.393	0.142	No
PLT 378-2 vs. JAM 355-1	4.234	15	7.072	<0.001	Do Not Test
PLT 378-2 vs. VNT 741-1	4.004	15	3.861	0.310	Do Not Test
PLT 378-2 vs. VNT 283-1	3.531	15	5.108	0.039	Do Not Test
PLT 378-2 vs. PTA 767-8	3.394	15	5.668	0.012	Do Not Test
PLT 378-2 vs. VNT 250-2	2.994	15	5.001	0.048	Do Not Test
PLT 378-2 vs. LES 358-4	2.508	15	4.189	0.196	Do Not Test
PLT 378-2 vs. VNT 896-4	1.954	15	3.264	0.588	Do Not Test
PLT 378-2 vs. HJT 349-3	1.225	15	1.547	0.999	Do Not Test
PLT 378-2 vs. OKA 970-3	0.910	15	1.520	0.999	Do Not Test
PLT 378-2 vs. VNT 366-2	0.729	15	1.217	1.000	Do Not Test
PLT 378-2 vs. MCQ 186-1	0.109	15	0.158	1.000	Do Not Test
MCQ 186-1 vs. SPEC 287-2	5.292	15	4.841	0.065	No
MCQ 186-1 vs. VNT 365-1	4.578	15	5.922	0.007	Do Not Test
MCQ 186-1 vs. BLB 331-2	4.447	15	4.068	0.234	Do Not Test
MCQ 186-1 vs. JAM 355-1	4.125	15	5.966	0.006	Do Not Test
MCQ 186-1 vs. VNT 741-1	3.894	15	3.563	0.442	Do Not Test
MCQ 186-1 vs. VNT 283-1	3.422	15	4.427	0.135	Do Not Test
MCQ 186-1 vs. PTA 767-8	3.284	15	4.750	0.077	Do Not Test
MCQ 186-1 vs. VNT 250-2	2.885	15	4.173	0.201	Do Not Test
MCQ 186-1 vs. LES 358-4	2.399	15	3.470	0.486	Do Not Test
MCQ 186-1 vs. VNT 896-4	1.845	15	2.669	0.851	Do Not Test
MCQ 186-1 vs. HJT 349-3	1.116	15	1.291	1.000	Do Not Test
MCQ 186-1 vs. OKA 970-3	0.801	15	1.158	1.000	Do Not Test
MCQ 186-1 vs. VNT 366-2	0.619	15	0.896	1.000	Do Not Test
VNT 366-2 vs. SPEC 287-2	4.673	15	4.506	0.118	Do Not Test
VNT 366-2 vs. VNT 365-1	3.958	15	5.725	0.011	Do Not Test
VNT 366-2 vs. BLB 331-2	3.827	15	3.691	0.383	Do Not Test
VNT 366-2 vs. JAM 355-1	3.505	15	5.854	0.008	Do Not Test
VNT 366-2 vs. VNT 741-1	3.275	15	3.158	0.641	Do Not Test
VNT 366-2 vs. VNT 283-1	2.803	15	4.053	0.239	Do Not Test
VNT 366-2 vs. PTA 767-8	2.665	15	4.451	0.130	Do Not Test
VNT 366-2 vs. VNT 250-2	2.266	15	3.784	0.342	Do Not Test
VNT 366-2 vs. LES 358-4	1.780	15	2.972	0.729	Do Not Test
VNT 366-2 vs. VNT 896-4	1.226	15	2.047	0.979	Do Not Test
VNT 366-2 vs. HJT 349-3	0.496	15	0.626	1.000	Do Not Test

VNT 366-2 vs. OKA 970-3	0.181	15	0.303	1.000	Do Not Test
OKA 970-3 vs. SPEC 287-2	4.491	15	4.331	0.158	Do Not Test
OKA 970-3 vs. VNT 365-1	3.777	15	5.463	0.019	Do Not Test
OKA 970-3 vs. BLB 331-2	3.646	15	3.516	0.464	Do Not Test
OKA 970-3 vs. JAM 355-1	3.324	15	5.552	0.016	Do Not Test
OKA 970-3 vs. VNT 741-1	3.094	15	2.983	0.724	Do Not Test
OKA 970-3 vs. VNT 283-1	2.621	15	3.791	0.339	Do Not Test
OKA 970-3 vs. PTA 767-8	2.484	15	4.148	0.209	Do Not Test
OKA 970-3 vs. VNT 250-2	2.084	15	3.481	0.481	Do Not Test
OKA 970-3 vs. LES 358-4	1.598	15	2.669	0.851	Do Not Test
OKA 970-3 vs. VNT 896-4	1.044	15	1.744	0.995	Do Not Test
OKA 970-3 vs. HJT 349-3	0.315	15	0.397	1.000	Do Not Test
HJT 349-3 vs. SPEC 287-2	4.177	15	3.602	0.423	Do Not Test
HJT 349-3 vs. VNT 365-1	3.462	15	4.006	0.256	Do Not Test
HJT 349-3 vs. BLB 331-2	3.331	15	2.873	0.773	Do Not Test
HJT 349-3 vs. JAM 355-1	3.009	15	3.799	0.336	Do Not Test
HJT 349-3 vs. VNT 741-1	2.779	15	2.397	0.927	Do Not Test
HJT 349-3 vs. VNT 283-1	2.306	15	2.669	0.851	Do Not Test
HJT 349-3 vs. PTA 767-8	2.169	15	2.738	0.826	Do Not Test
HJT 349-3 vs. VNT 250-2	1.769	15	2.234	0.957	Do Not Test
HJT 349-3 vs. LES 358-4	1.283	15	1.620	0.998	Do Not Test
HJT 349-3 vs. VNT 896-4	0.729	15	0.921	1.000	Do Not Test
VNT 896-4 vs. SPEC 287-2	3.447	15	3.324	0.559	Do Not Test
VNT 896-4 vs. VNT 365-1	2.733	15	3.952	0.275	Do Not Test
VNT 896-4 vs. BLB 331-2	2.602	15	2.509	0.900	Do Not Test
VNT 896-4 vs. JAM 355-1	2.280	15	3.808	0.332	Do Not Test
VNT 896-4 vs. VNT 741-1	2.049	15	1.976	0.985	Do Not Test
VNT 896-4 vs. VNT 283-1	1.577	15	2.281	0.950	Do Not Test
VNT 896-4 vs. PTA 767-8	1.439	15	2.404	0.926	Do Not Test
VNT 896-4 vs. VNT 250-2	1.040	15	1.737	0.996	Do Not Test
VNT 896-4 vs. LES 358-4	0.554	15	0.925	1.000	Do Not Test
LES 358-4 vs. SPEC 287-2	2.893	15	2.790	0.806	Do Not Test
LES 358-4 vs. VNT 365-1	2.179	15	3.151	0.644	Do Not Test
LES 358-4 vs. BLB 331-2	2.048	15	1.975	0.985	Do Not Test
LES 358-4 vs. JAM 355-1	1.726	15	2.882	0.769	Do Not Test
LES 358-4 vs. VNT 741-1	1.495	15	1.442	0.999	Do Not Test
LES 358-4 vs. VNT 283-1	1.023	15	1.480	0.999	Do Not Test
LES 358-4 vs. PTA 767-8	0.885	15	1.479	0.999	Do Not Test
LES 358-4 vs. VNT 250-2	0.486	15	0.812	1.000	Do Not Test
VNT 250-2 vs. SPEC 287-2	2.407	15	2.321	0.943	Do Not Test
VNT 250-2 vs. VNT 365-1	1.693	15	2.448	0.916	Do Not Test
VNT 250-2 vs. BLB 331-2	1.562	15	1.506	0.999	Do Not Test
VNT 250-2 vs. JAM 355-1	1.240	15	2.071	0.977	Do Not Test
VNT 250-2 vs. VNT 741-1	1.009	15	0.973	1.000	Do Not Test
VNT 250-2 vs. VNT 283-1	0.537	15	0.777	1.000	Do Not Test
VNT 250-2 vs. PTA 767-8	0.399	15	0.667	1.000	Do Not Test
PTA 767-8 vs. SPEC 287-2	2.008	15	1.936	0.987	Do Not Test
PTA 767-8 vs. VNT 365-1	1.293	15	1.870	0.991	Do Not Test
PTA 767-8 vs. BLB 331-2	1.162	15	1.121	1.000	Do Not Test
PTA 767-8 vs. JAM 355-1	0.840	15	1.404	1.000	Do Not Test

PTA 767-8 vs. VNT 741-1	0.610	15	0.588	1.000	Do Not Test
PTA 767-8 vs. VNT 283-1	0.138	15	0.199	1.000	Do Not Test
VNT 283-1 vs. SPEC 287-2	1.870	15	1.711	0.996	Do Not Test
VNT 283-1 vs. VNT 365-1	1.156	15	1.495	0.999	Do Not Test
VNT 283-1 vs. BLB 331-2	1.025	15	0.938	1.000	Do Not Test
VNT 283-1 vs. JAM 355-1	0.703	15	1.017	1.000	Do Not Test
VNT 283-1 vs. VNT 741-1	0.473	15	0.432	1.000	Do Not Test
VNT 741-1 vs. SPEC 287-2	1.398	15	1.044	1.000	Do Not Test
VNT 741-1 vs. VNT 365-1	0.683	15	0.625	1.000	Do Not Test
VNT 741-1 vs. BLB 331-2	0.552	15	0.413	1.000	Do Not Test
VNT 741-1 vs. JAM 355-1	0.230	15	0.222	1.000	Do Not Test
JAM 355-1 vs. SPEC 287-2	1.167	15	1.126	1.000	Do Not Test
JAM 355-1 vs. VNT 365-1	0.453	15	0.655	1.000	Do Not Test
JAM 355-1 vs. BLB 331-2	0.322	15	0.310	1.000	Do Not Test
BLB 331-2 vs. SPEC 287-2	0.845	15	0.631	1.000	Do Not Test
BLB 331-2 vs. VNT 365-1	0.131	15	0.120	1.000	Do Not Test
VNT 365-1 vs. SPEC 287-2	0.715	15	0.654	1.000	Do Not Test

A result of "Do Not Test" occurs for a comparison when no significant difference is found between two means that enclose that comparison. For example, if you had four means sorted in order, and found no difference between means 4 vs. 2, then you would not test 4 vs. 3 and 3 vs. 2, but still test 4 vs. 1 and 3 vs. 1 (4 vs. 3 and 3 vs. 2 are enclosed by 4 vs. 2: 4 3 2 1). Note that not testing the enclosed means is a procedural rule, and a result of Do Not Test should be treated as if there is no significant difference between the means, even though one may appear to exist.
