# Supplementary material for: Solanum venturii, a suitable model system for virus-induced gene silencing studies in potato reveals StMKK6 as an important player in plant immunity
Source: Plant Methods. 2016 May 20;12:29. doi: 10.1186/s13007-016-0129-3 (PMC4875682; doi:10.1186/s13007-016-0129-3)
Supplement: Supplementary file 6 — 10.1186/s13007-016-0129-3 Number of infected plants at each time point in StMKK6 silenced and empty vector treated plants for two different experiments. The table shows the actual number of plants, which were used to calculate cumulative percentage of systemically infected plants after TRV-based VIGS in relation to days post inoculation, as shown in Fig. 4. [file 13007_2016_129_MOESM6_ESM.pdf]

Additional file 6: Number of infected plants at each time point in StMKK6 silenced and empty vector treated plants for two different experiments.

The table shows the actual number of plants, which were used to calculate cumulative percentage of systemically infected plants after TRV-based VIGS in relation to days post inoculation, as shown in Figure 4.

	Experiment 1		Experiment 2	
dpi	St*MKK6* silenced	Empty vector	St*MKK6* silenced	Empty vector
12	1	0		
14	1	0		
16			1	0
18	1	0		
20			3	0
21	4	1		
23			5	2
24	4	1		
26			6	3
Total replicates	5	5	6	4
